# Mapping *Aedes aegypti* (Diptera: Culicidae) and *Aedes albopictus* Vector Mosquito Distribution in Brownsville, TX

**DOI:** 10.1093/jme/tjz132

**Published:** 2019-08-10

**Authors:** Mark H Myer, Chelsea M Fizer, Kenneth R Mcpherson, Anne C Neale, Andrew N Pilant, Arturo Rodriguez, Pai-Yei Whung, John M Johnston

**Affiliations:** 1 ORISE U.S. Environmental Protection Agency, Athens, GA; 2 ORAU U.S. Environmental Protection Agency, Durham, NC; 3 U.S. Environmental Protection Agency, Dallas, TX; 4 U.S. Environmental Protection Agency, Durham, NC; 5 Public Health Department, Brownsville, TX; 6 U.S. Environmental Protection Agency, Athens, GA

**Keywords:** *Aedes*, Bayesian, statistical model, weather, mosquito control

## Abstract

*Aedes* mosquitoes are vectors of several emerging diseases and are spreading worldwide. We investigated the spatiotemporal dynamics of *Aedes aegypti* (Linnaeus) and *Aedes albopictus* (Skuse) mosquito trap captures in Brownsville, TX, using high-resolution land cover, socioeconomic, and meteorological data. We modeled mosquito trap counts using a Bayesian hierarchical mixed-effects model with spatially correlated residuals. The models indicated an inverse relationship between temperature and mosquito trap counts for both species, which may be due to the hot and arid climate of southern Texas. The temporal trend in mosquito populations indicated *Ae. aegypti* populations peaking in the late spring and *Ae. albopictus* reaching a maximum in winter. Our results indicated that seasonal weather variation, vegetation height, human population, and land cover determine which of the two *Aedes* species will predominate.

In the last two decades, outbreaks of previously rare diseases have shifted the focus of U.S. mosquito vector control from *Culex* mosquitoes, the vector of malaria, and West Nile virus, to the genus *Aedes*, which transmits many arboviruses of emerging concern including chikungunya, Zika virus, and dengue fever. Global trade, especially in used tires, has led to the nearly worldwide dispersal of vector mosquitoes including *Aedes aegypti* (Linnaeus) and *Aedes albopictus* (Skuse) (Sprenger 1987). Human movement patterns, increased urbanization, and changes in climate are predicted to expand the fraction of the global population that are at risk from *Ae. aegypti* and *Ae. albopictus* mosquitoes to 49% by 2050 (Kraemer et al. 2019). There had been no locally transmitted cases of dengue fever in the United States for more than 50 yr until the early 2000s, when cases were detected in Hawaii (Effler et al. 2005), Texas (Centers for Disease Control and Prevention [CDC] 2007), and Florida (Centers for Disease Control and Prevention [CDC] 2010). Autochthonous Zika transmission was not reported in the Western hemisphere until 2015 when cases were detected in South America and Central America and subsequently in the United States beginning in 2016 (Pan American Health Organization 2017). Chikungunya spread with similar rapidity, with first detection in 2013 in Saint Martin leading to nearly 1 million cases in the Western hemisphere in the subsequent year and quickly spreading throughout the Americas, including autochthonous cases in Florida (Hamer and Chen 2014). None of these diseases have an effective vaccine, and prevention efforts focus on control of *Aedes* mosquitoes.

In the southern United States, the prevalent *Aedes* species are *Ae. aegypti* and *Ae. albopictus*. *Aedes aegypti* have been present in the Western hemisphere since the 16th century when they were introduced by trans-Atlantic trade (Chadee et al. 1998), whereas *Ae. albopictus* were first detected in the United States in Texas in 1985 and probably arrived in used tires from Asia (Sprenger and Wuithiranyagool 1986, Hawley et al. 1987). Meteorological factors associated with *Aedes* presence include air temperature between approximately 15 and 30°C (Alto and Juliano 2001, Yang et al. 2009, Brady et al. 2013) and recent precipitation, which provides the standing water necessary for egg and larval development. Land cover factors influence the distribution of adult *Aedes* mosquitoes. *Aedes albopictus* adults are generally exophilic, preferring to rest outdoors on plant cover including trees and shrubs (Delatte et al. 2010, Samson et al. 2013), whereas *Ae. aegypti* are cosmopolitan and will rest close to human habitation or even indoors (Perich et al. 2000, Dzul-Manzanilla et al. 2016). Socioeconomic influences on *Aedes* mosquito populations are generally related to urbanization, including impervious cover, housing age, income, and urban development (Rey et al. 2006, Landau and van Leeuwen 2012, Sallam et al. 2017). The two species are known to compete for resources as larvae, with outcome dependent on environmental conditions (Ho et al. 1989, Lounibos et al. 2002, Braks et al. 2004).

A systematic review of *Ae. aegypti* and *Ae. albopictus* distribution modeling concluded that a wide range of variables was needed to predict the distribution of these species (Sallam et al. 2017). The review highlighted heterogeneity in observed effects of commonly used predictors that reinforced the need to customize models in areas of interest. We worked with the Brownsville Public Health Department to model *Ae. aegypti* and *Ae. albopictus* populations across their downtown area based on sampling from 80 mosquito traps across the city in 2017, and a range of ecological, socioeconomic, and meteorological predictors, with the goal of creating a risk map that would help target vector control resources.

## Methods

### Study Area

Brownsville, TX, is a city in the United States located at the southernmost tip near the Rio Grande river, at the coordinates 25°55′49″N 97°29′4″W. It has a population of approximately 183,299 people and a median annual household income of $35,636 (U.S. Census Bureau 2018). Brownsville is located near the nexus of two Köppen climate regions: humid subtropical (Cfa) to the east and hot semiarid (BSh) to the west and south (Peel et al. 2007). Mean annual rainfall is 697 mm, with a maximum in September and a dry season in November through April. Monthly mean high temperatures range from a high of 34.7°C in August to a low of 10.9°C in February, with a long hot summer and a short mild winter. Weather patterns are dominated by airflow from the Gulf of Mexico to the east, with intermittent weather patterns bringing dry and hot tropical air from Mexico to the southwest (Crescenti 1997).

### Mosquito Trapping and Identification

**Fig. 1.**
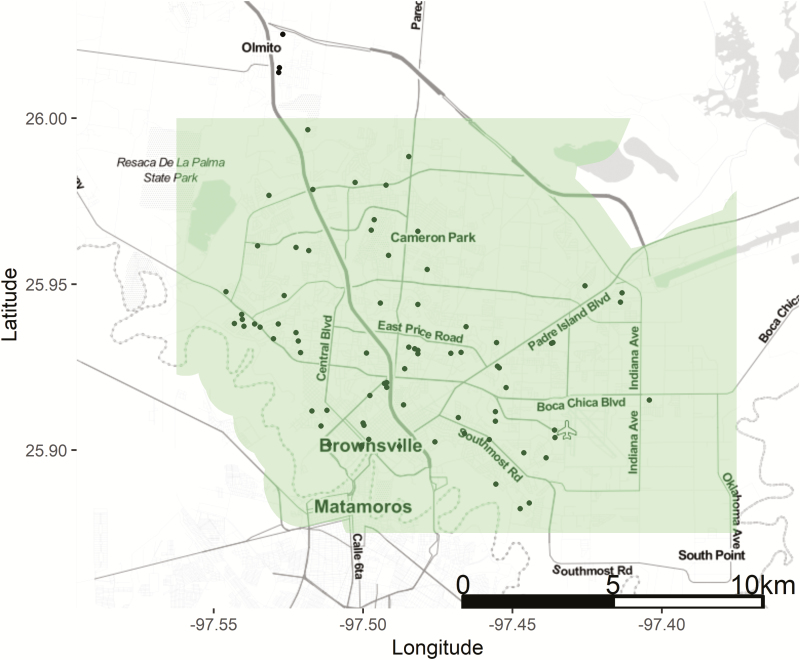
Locations of BT-Sentinel mosquito traps in Brownsville, TX. Trap locations are shown as circles. The shaded area represents the City of Brownsville priority boundary for vector control efforts.

We obtained mosquito trap data from the Brownsville Public Health Department from 80 locations in the metropolitan area from January to December 2017 (*n* = 5,367 observations). BG-Sentinel traps (Biogents AG, Regensburg, Bavaria, Germany) were deployed throughout the study area (Fig. 1). Because of land ownership concerns and personnel constraints, traps were not placed randomly or sampled equally throughout the year (Supp Figs. 1 and 2 [online only]). We discarded trap sites with fewer than 30 observations (Supp Fig. 3 [online only]), leaving 51 trap sites and a total of 5,079 observations for analysis. The mean number of observations at the remaining trap sites was 100. The mean distance between trap sites was approximately 5.9 km (SD 3.4 km). Trap contents were shipped to the Texas Department of State Health Services, Zoonosis Control Branch in Austin, TX, for identification. Taxonomists identified the mosquito trap contents to the species level using the Darsie and Ward’s (2005) taxonomic keys and reported the count of each mosquito species present. Mosquitoes that had morphological damage to key characters were not counted if identification to the species level was in doubt. The number of *Ae. aegypti* and *Ae. albopictus* individuals counted in each sample (mosquito trap count) were the dependent variables for our models.

### Independent Variable Collection

We obtained land cover, meteorological data, vegetation height, housing age and value, and population data from several sources (Table 1) and summarized these in 0.1-km-radius circular buffers around the trap sites using Arc ModelBuilder and the *Spatial Analyst* toolbox in ArcGIS 10.6. The radius was chosen as representative of the observed dispersal range of *Ae. aegypti* mosquitoes in comparable climate zones (Muir and Kay 1998, Harrington et al. 2001). Variables were centered and scaled by subtracting the mean and dividing by the standard deviation. On this scale, a value of zero represents the mean, and the units are standard deviations from the mean.

**Table 1.** Independent variables considered for inclusion in mosquito models

Variable	Units	Source
Water cover	Proportion of buffer area	EnviroAtlas MULC^*^a^*^
Impervious cover	Proportion of buffer area	EnviroAtlas MULC
Soil cover	Proportion of buffer area	EnviroAtlas MULC
Tree cover	Proportion of buffer area	EnviroAtlas MULC
Shrub cover	Proportion of buffer area	EnviroAtlas MULC
Grass cover	Proportion of buffer area	EnviroAtlas MULC
Vegetation height	Mean height in meters	IBWC nDSM^*^b^*^
Population density	Mean persons per kilometer	EnviroAtlas^*^c^*^
Housing value	Mean dollar value	Cameron County, TX Land Parcel Data^*^d^*^
Proportion houses built before 1939	Proportion of residential homes	Cameron County, TX Land Parcel Data
Proportion houses built before 1949	Proportion of residential homes	Cameron County, TX Land Parcel Data
Proportion houses built before 1959	Proportion of residential homes	Cameron County, TX Land Parcel Data
Proportion houses built before 1969	Proportion of residential homes	Cameron County, TX Land Parcel Data
Proportion houses built before 1979	Proportion of residential homes	Cameron County, TX Land Parcel Data
Proportion houses built before 1989	Proportion of residential homes	Cameron County, TX Land Parcel Data
Proportion houses built before 1999	Proportion of residential homes	Cameron County, TX Land Parcel Data
Proportion houses built before 2009	Proportion of residential homes	Cameron County, TX Land Parcel Data
Proportion houses built 2010 and newer	Proportion of residential homes	Cameron County, TX Land Parcel Data
Temperature	Degree Celsius	Daymet^*^e^*^
Precipitation	Millimeters	Daymet
Shortwave irradiance	Watts per square meter	Daymet
Humidity	Pascals of water partial vapor pressure	Daymet

^*a*^Meter-scale urban land cover, https://www.epa.gov/enviroatlas, resolution 1 m.

^*b*^International Boundary and Water Commission normalized digital surface model, https://tnris.org/data-catalog/entry/ibwc-2011-70cm/, resolution 1 m.

^*c*^US Environmental Protection Agency EnviroAtlas, https://www.epa.gov/enviroatlas, resolution 1 km.

^*d*^Provided by Cameron County, TX.

^*e*^Daily surface weather and climatological summaries, https://daymet.ornl.gov, resolution 1 km.

Our choice of independent variables was guided by the availability of data to cover the entirety of the study area and a survey of the literature to establish mechanistic links with mosquito populations. Precipitation and temperature have well-established links with mosquito populations (Alto and Juliano 2001), and we also considered humidity and shortwave irradiance. Humidity can affect the relative abundance of competing mosquito species, as their eggs have differing tolerance to desiccation (Juliano et al. 2002), and variations in temperature and humidity have been shown to affect the reproductive cycle of *Ae. aegypti* (Costa et al. 2010, Chaves et al. 2012). Shortwave irradiance is a correlate of sunlight exposure and photosynthetically active radiation (Britton and Dodd 1976), and the diurnal surface water temperature stability of small water bodies, where mosquito larvae are found, is strongly affected by radiant energy input (Jacobs et al. 1998). Mosquito larval development times correlate more strongly with water temperature than with air temperature (Asare et al. 2016). We obtained air temperature, humidity, and shortwave irradiance data as mean daily values, whereas precipitation was a daily total.

Land cover is widely used to predict mosquito abundance and disease risk in urban ecosystems, and the availability of a 1-m resolution data set for Brownsville, TX, led us to include it in our independent variable set as fine-scale land cover data are particularly effective in urban mosquito modeling (Landau and van Leeuwen 2012). Because our study area is largely urban, we obtained housing age and value data from the Cameron County, Texas cadastral data set. Older housing and low socioeconomic status (SES) have been associated with *Aedes* mosquito populations (Dowling et al. 2013, Sallam et al. 2017) and mosquito-borne disease risk (Ruiz et al. 2004). The link between SES and mosquito populations is not well understood and studies have had contradictory results, with recent research indicating higher mosquito populations in high-SES areas (Becker et al. 2014). These differences may result from differing sources of standing water for mosquito oviposition, with disused containers predominating in low-SES communities and managed containers such as landscaping features predominating in high-SES communities. Although overall prevalence of standing water was higher in high-SES communities, a greater number of mosquito larvae were found in disused containers in low-SES communities. We included vegetation height derived from LiDAR for areas of land cover that were classified as predominantly plants (Trees, Grass, and Shrub classifications in our data set) because *Aedes* mosquitoes have been shown to prefer shaded areas with overhanging vegetation for oviposition. Tree or shrub cover stabilizes larval habitat temperatures and provides a source of organic detritus for larval feeding (Barrera et al. 2006a,b).

### Data Analysis

We selected independent variables for model development with Bayesian feature selection, using the package spikeSlabGAM in R 3.5.1 (Schiepl et al. 2012, R Core Team 2018). The method involves fitting many Bayesian additive models using Markov chain Monte Carlo, then ranking independent variables on their posterior probability for inclusion in the best-fitting model. An in-depth explanation of spike-and-slab variable selection can be found in George and McCulloch (1997). For each mosquito species, a separate regression was run with all 22 potential variables, with rankings based on their posterior inclusion probability in the final model. From the variables with the highest posterior inclusion probabilities, we chose the six with the largest estimated scaled effect sizes. We limited variables for model parsimony and interpretability. We tested weather variables with 0 lag, 1-wk lag time (hereafter referred to as Lag 1), and 2-wk lag time (Lag 2), representing the daily value for the day of the observation, 7 d previously, and 14 d previously, respectively, but only considered the lag time with the largest effect size. We used correlation plots (Supp Fig. 4 [online only]) for the six variables chosen for each species to examine intervariable relationships.

We chose hierarchical mixed-effects modeling as our analysis method because it is robust to unbalanced longitudinal data (Laird and Ware 1982). We modeled mosquito trap counts using hierarchical mixed-effects Bayesian models, with the package *R-INLA* (Rue et al. 2009). This approach to computing approximate spatially correlated effects for geospatial data enables analysis of large spatial data sets without requiring a powerful computer (Lindgren et al. 2011). We fit a Poisson generalized linear model to each *Aedes* species data set with no spatial effect and calculated the overdispersion statistic based on Pearson residuals. We then fit zero-inflated Poisson models to each data set, to determine whether a zero-inflated model would result in better fit based on the large number of zero mosquito count observations in the data (*Ae. aegypti n* = 898, *Ae. albopictus n* = 4,265). We then fit a negative binomial model to each data set to correct for overdispersion. Of these three options, the model with the lowest deviance information criterion (DIC) value, an indicator of model fit, was chosen. For both species, the best model was the negative binomial. We added a spatially correlated random effect to the model to account for relationships among proximate observations. For pairs of variables that were correlated (|Spearman’s *ρ*|>0.5), we fit a model with one variable dropped, then the other, and with an interaction term.

In a Bayesian model, prior beliefs about the variables and parameters must be specified. We addressed prior selection by using the penalized-complexity (PC) priors suggested by Fuglstad et al. (2018), which generate weakly informative priors. For the prior on spatial correlation, we decided that it was reasonable to specify that based on the dispersal range of *Aedes* mosquitoes, it was unlikely that observations farther apart than half the width of the study area (10 km) would be correlated and that the spatial effect would likely be relatively small compared with the effects of the independent variables (p[spatial correlation range < 10 km] = 0.95, p[spatial effect standard deviation > 0.5] = 0.05). We used default PC priors for the independent variables, a zero-inflation parameter, and an overdispersion parameter.

We evaluated model fit by conducting a simulation study, sampling 1,000 simulated parameter sets from the posteriors of the model then calculating 1,000 sets of simulated mosquito counts. We plotted a histogram of the simulated results with 95% prediction intervals against the observed mosquito counts. We considered a model that captures the observed counts within the 95% prediction interval a good predictive fit. We conducted residual analysis by computing the scaled residuals for each of the 1,000 simulations and comparing them to the residuals of the observed data.

We plotted the spatially correlated effect of the model across the Brownsville metropolitan area to evaluate hotspots of mosquito activity and visualize the spatial relationships among mosquito observations. Because predictions of the spatial effect are not made at every point within the study area, we used *R-INLA*’s built-in projection function to interpolate the model results across the metropolitan area for visualization.

We used the best models to make weekly mosquito count predictions throughout 2017 for the Brownsville metropolitan area using 1 km^2^ gridded weekly weather data from Daymet (Thornton et al. 2016). Risk maps were produced for the study area extent at the resolution of the meteorological variables from Daymet (1 km^2^).

## Results

### Dependent Variable Description

**Fig. 2.**
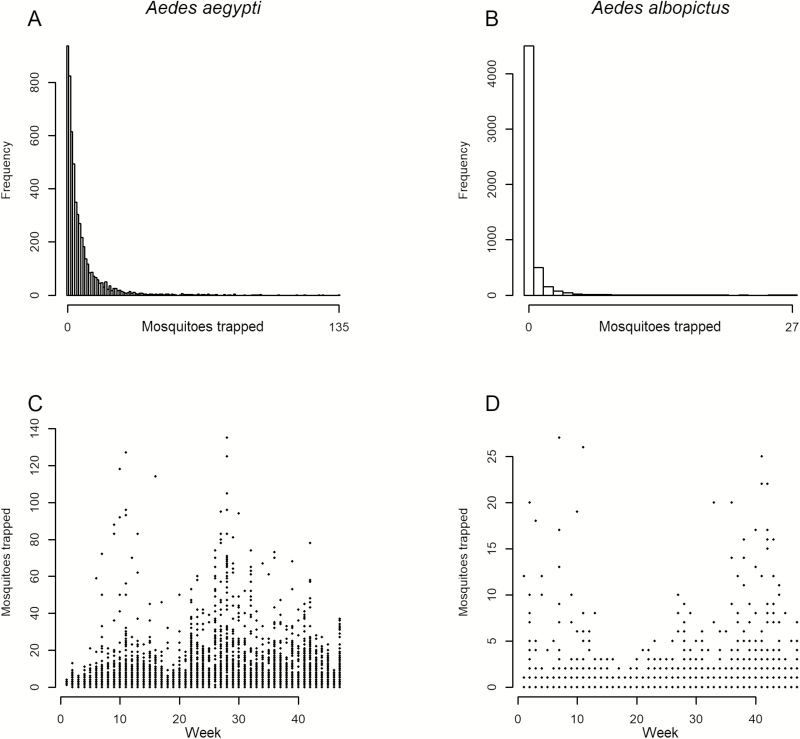
Number of *Aedes aegypti* and *Aedes albopictus* trapped by frequency of count observed (A and B) and by ordinal week (C and D). *Aedes aegypti* counts had a maximum of 135 mosquitoes and a zero-excluded median of 4. *Aedes albopictus* counts were lower than *Ae. aegypti* counts, with a maximum count of 27 mosquitoes per trap and a zero-excluded median of 1.

*Aedes aegypti* and *Ae. albopictus* trap counts (Fig. 2A and B), suggested a Poisson distribution. The most frequent response was zero, for either species in a trap; 17.7% of the traps had zero *Ae.* aegypti and 84.0% of the traps had zero *Ae. albopictus*. *Aedes aegypti* counts peaked in mid-July, with a secondary mode in the first week of March (Fig. 2C); trap counts were generally higher in the second half of the year. *Aedes albopictus* counts were generally higher before March and after August, with fewer in the spring and summer (Fig. 2D).

### Independent Variable Selection

The Bayesian spike-and-slab selection yielded the following independent variables for *Ae. aegypti*: tree cover, water cover, proportion houses built before 1979, shortwave irradiance, humidity, and lag 1 temperature. For *Ae. albopictus*, the variables were impervious cover, grass cover, proportion houses built before 1969, population density, lag 2 humidity, and lag 1 temperature.

The correlation plots for each species’ set of independent variables showed that temperature was positively associated with humidity (Spearman’s ρ = 0.61). Among nonweather variables, impervious cover was negatively associated with grass cover (ρ = −0.67). Because we were interested in the ecological questions associated with these variables, we left them in the model and adjusted our interpretation to consider the possible effects of multicollinearity. We fit models with each pair of correlated variables removed and with interaction terms included (Supp Tables 1–12 [online only]). For both mosquito species, the interaction terms were significant, changed the interpretation of the model, and improved DIC.

### Model Selection

Comparison of Poisson, zero-inflated Poisson, and negative binomial models for each mosquito species by DIC (Table 2) showed the negative binomial model to be the best fit for both species. The overdispersion statistic was greater than one for the negative binomial models, indicating that the expected variance was greater than the mean and that the negative binomial model was appropriate (*Ae. aegypti* overdispersion statistic 1.27 [95% CI = 1.22–1.33], *Ae. albopictus* overdispersion statistic 4.18 [95% CI = 3.71–4.72]). Adding a spatially correlated random effect improved both models according to DIC.

**Table 2.** Goodness-of-fit measures for model types by species

Species			
*Aedes aegypti*		*Aedes albopictus*	
Model	DIC	Model	DIC
Poisson	61,671.44	Poisson	9,256.79
Zero-inflated Poisson	55,585.97	Zero-inflated Poisson	7,814.99
Negative binomial	30,375.47	Negative binomial	6,891.11
Negative binomial + spatial effect	29,053.57	Negative binomial + spatial effect	6,306.60

DIC (deviance information criterion).

### Model Results and Validation

The negative binomial models with spatially correlated random effects (Table 3) showed statistically important (a Bayesian statistical statement referring to independent variables with a 95% credible interval around the coefficient that does not encompass zero) relationships between several of the independent variables and the mosquito count response. For *Ae. aegypti*, we found important positive relationships between mosquito count and tree cover (log incidence rate ratio 0.34; 95% CI = 0.15–0.53), humidity (0.46; 0.41–0.51), and shortwave irradiance (0.52; 0.46–0.57), and important negative relationships for lag 1 temperature (−0.21; −0.26 to −0.16) and the interaction between humidity and lag 1 temperature (−0.08; −0.12 to −0.04). Water cover and the proportion of houses built before 1979 were not found to be important. For *Ae. albopictus*, we found an important positive relationship between mosquito count and lag 2 humidity (log incidence rate ratio 0.55; 95% CI = 0.44–0.65), and important negative relationships with impervious cover (−0.41; −0.80 to −0.02), grass cover (−0.25; −0.65 to −0.18), population density (−0.80; −1.16 to −0.44), and lag 1 temperature (−0.50; −0.59 to −0.41). The proportion of houses built before 1969 was not important. The spatial effects had a short correlation range, 0.9 km (95% CI = 0.5–1.5) for *Ae. aegypti* and 1.0 km (95% CI = 0.6–1.7) for *Ae. albopictus* (Supp Fig. 6 [online only]). This indicates that mosquito count observations are related to one another at relatively short distances (<2 km) and that observations from trap sites farther away were probably uncorrelated.

**Table 3.** Summary of best-fitting model coefficients and parameters by species

Species					
*Aedes aegypti*			*Aedes albopictus*		
Model selected					
Negative binomial			Negative binomial		
Variable	Coefficient (IRR)		Variable	Coefficient (IRR)	
	Mean	95% CI		Mean	95% CI
Intercept	1.59	1.33 to 1.83	Intercept	−1.50	−1.97 to −1.04
**Trees**	0.34	0.15 to 0.53	**Impervious**	−0.41	−0.80 to −0.02
Water	−0.01	−0.15 to 0.12	**Grass**	−0.25	−0.65 to −0.18
Built before 1979	0.09	−0.04 to 0.22	Built before 1969	0.00	−0.18 to −0.18
**Humidity** ^*a*^	0.46	0.41 to 0.51	**Population density**	−0.80	−1.16 to −0.44
**Shortwave irradiance** ^*a*^	0.52	0.46 to 0.57	**Lag 2 humidity** ^*a*^	0.55	0.44 to 0.65
**Lag 1 temperature** ^*a*^	−0.21	−0.26 to −0.16	**Lag 1 temperature** ^*a*^	−0.50	−0.59 to −0.41
**Lag 1 temperature × humidity**	−0.08	−0.12 to −0.04	**Impervious × grass**	0.42	0.11 to 0.75
			**Lag 1 temperature × lag 2 humidity**	−0.13	−0.23 to −0.04

Parameter			Parameter		
Overdispersion	0.92	0.72 to 0.97	Overdispersion	2.27	2.00 to 2.63
Spatial range	0.9 km	0.5 to 1.5 km	Spatial range	1.0 km	0.6 to 1.7 km

Statistically important relationships are in bold text. IRR (log incidence rate ratio); CI (credible interval).

^*a*^Daily mean value.

**Fig. 3.**
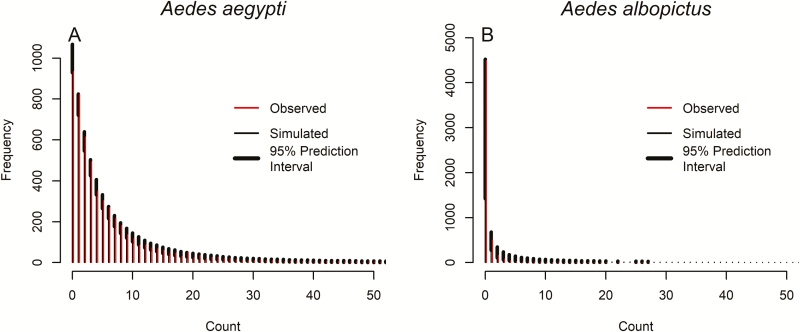
Simulation study results of 1,000 simulated data sets from 1,000 posterior samples for *Aedes aegypti* (A) and *Aedes albopictus* (B). An observed count, shown as a thin line, within the 95% prediction interval, shown as a thick band, is an indicator of good model prediction.

The simulation histograms indicated that the models predicted the distribution of mosquito counts in the sample adequately, with 95% prediction intervals encompassing the observed counts (Fig. 3A and B). For *Ae. albopictus*, the observed number of zeroes in the data set is near the top of the 95% predictive interval, which indicates that our model is likely to underpredict the number of zeroes. Residual studies to test overprediction or underprediction revealed no patterns in the scaled residuals for *Ae. aegypti*, but the scaled residuals for *Ae. albopictus* indicated model underprediction.

**Fig. 4.**
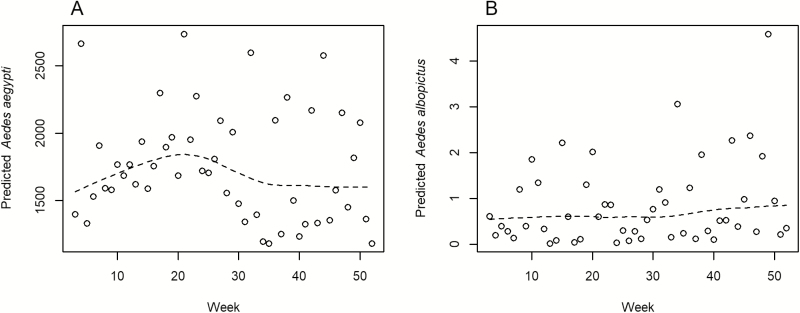
Predicted numbers of mosquitoes trapped by week for *Aedes aegypti* (A) and *Aedes albopictus* (B). The dotted line represents a loess curve fitted to the data to aid in visualization of the trend in mosquitoes trapped over time. Predicted *Ae. aegypti* counts peaked in late May. Predicted *Ae. albopictus* counts were low throughout the year but reached their greatest value in early December.

**Fig. 5.**
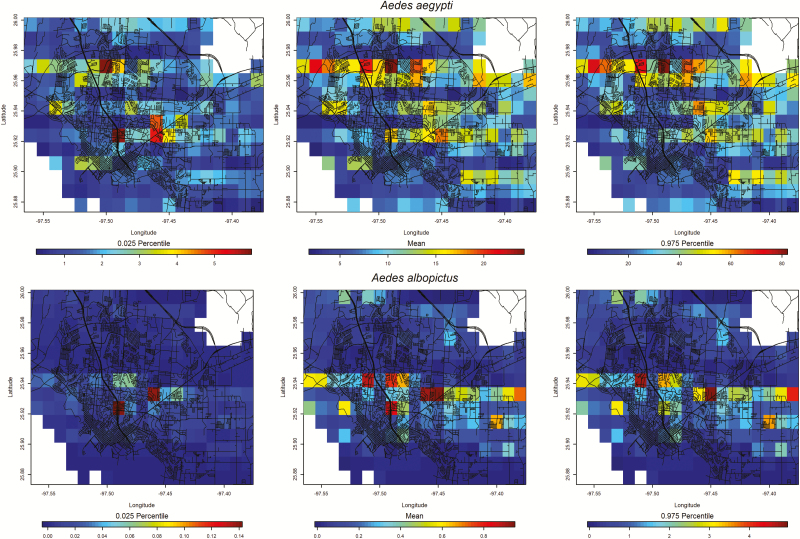
Predicted values for mosquito trap counts across the Brownsville metropolitan area during the week of highest expected incidence in 2017, with 95% predictive intervals. For *Aedes aegypti*, the highest trap counts were predicted on the week of 22–28 May, whereas for *Ae. albopictus*, the highest counts were predicted from 4 to 10 December.

The model predictions showed that peak *Ae. aegypti* counts were expected from 22 to 28 May (Fig. 4A) and that peak *Ae. albopictus* counts were expected from 4 to 10 December, although predicted counts were low throughout the year (Fig. 4B). The number of predicted *Ae. aegypti* was nearly three orders of magnitude greater than *Ae. albopictus* predictions at all time points. A prediction map was created for each species’ peak abundance week (Fig. 5). The northern part of the city above latitude 25.96 had greater *Ae. aegypti* counts, whereas the central part of the city near latitude 25.94 contained the greater *Ae. albopictus* counts.

## Discussion

Our analysis of the data from the *Aedes* mosquito vector surveillance program in Brownsville, TX, indicated that predictors associated with *Aedes aegypti* were largely different from those associated with *Ae. albopictus*, although both species had similar responses to meteorological variables. The number of *Ae. aegypti* predicted was greater than the number of *Ae. albopictus* at all time points, which is consistent with the observed surveillance data. Because of year-round warm temperatures, southern Texas is an area of elevated suitability for *Ae. aegypti*, which may explain this trend (Monaghan et al. 2016). A meta-analysis of 351 laboratory survivorship studies found that temperature affects interspecific competition between the two species and that although it had lower longevity at all temperatures tested, *Ae. aegypti* can tolerate a wider range of temperatures (Brady et al. 2013). We found that *Ae. albopictus* counts were relatively greater in the cooler months of the year. Similarly, in a study comparing container occupancy by the two species in Florida, high temperatures led to greater mortality in *Ae. albopictus* eggs whereas *Ae. aegypti* survivorship was unaffected (Juliano et al. 2002). The main effect of temperature, which in these models represents the effect of temperature when humidity is at its average, was negatively associated with both *Ae. aegypti* and *Ae. albopictus* counts, with a higher magnitude for *Ae. albopictus.* Brownsville experiences temperatures exceeding 30°C for more than half of the year, and these high temperatures have been found to favor *Ae. aegypti* in interspecific competition, with a stronger effect in urban areas (Farjana et al. 2012). Our model included temperature lagged at 1 wk for both *Ae. aegypti* and *Ae. albopictus* and humidity lagged at 2 wk for *Ae. albopictus*, which reflects the time needed for changes in larval and pupal survival to reflect in adult mosquito counts. Perturbations in temperature and rainfall can have long-lasting effects on adult mosquito populations in *Aedes* mosquitoes, with lag times reported up to 10 wk in some cases for *Ae. aegypti* (Chaves et al. 2014).

For both species, the main effect of humidity was positively associated with trap counts, which was expected as *Aedes* mosquitoes are susceptible to desiccation at low humidity (Hylton 1967). Brownsville experiences periodic episodes of dry air blowing from arid regions to the west, which result in lowered humidity and may limit mosquito populations by causing dehydration at all life stages and evaporating sources of fresh water for eggs and larvae. The magnitude of the effect was similar for both species, despite reports that *Ae. albopictus* eggs are more susceptible to desiccation than *Ae. aegypti* (Juliano et al. 2002). In both species, the interaction between humidity and temperature was negative, indicating that at higher temperature the effect of humidity decreases, and vice versa. The importance of humidity seen in both species may help explain the lower baseline abundance of *Ae. albopictus* in Brownsville despite the proximity of the city to the initial *Ae. albopictus* introduction site in the United States. Shortwave irradiance was associated with increased *Ae. aegypti* counts. *Aedes aegypti* larvae are negatively affected by large diurnal temperature swings and have higher adult reproductive output when water temperature remains stable (Carrington et al. 2013). Our results suggest that in hot areas of the southern United States with seasonal patterns of humidity, *Ae. aegypti* enjoys a competitive advantage over *Ae. albopictus* due to its greater hardiness to high temperature and desiccation.

*Aedes aegypti* abundance was positively associated with trees, probably due to increased larval productivity in shaded water bodies. Tree cover provides shade for shallow water bodies that function as *Ae. aegypti* larval habitat, protecting larvae from the harmful effects of direct sunlight and helping to keep temperatures stable (Barrera et al. 2006b). Fallen leaves also provide a source of organic detritus for larvae to feed on (Barrera et al. 2006a). *Aedes albopictus* counts were inversely related to indicators of human habitation, including impervious cover, grass cover, and human population density. The interaction between impervious cover and grass was positive and important, which indicates that the negative association of the presence of one land cover type with *Ae. albopictus* abundance was reduced by the presence of the other. There is a negative correlation between impervious cover and grass (Supp Fig. 5 [online only]) because the two land cover types are zero-sum: paving land reduces the amount of vegetative cover. The positive interaction term then represents the reduction in grass cover when impervious cover increases, and vice versa, and areas with both grass and impervious cover experience a smaller mediating effect on *Ae. albopictus* counts than areas with each land cover type alone. The associations between vegetation type and observed *Ae. albopictus* and *Ae. aegypti* abundance may be the result of organic detritus preferences: a study of larval interspecific competition between the two species in the presence of four detritus types (oak leaves, pine needles, grass, or insect carcasses) found that grass was the only substrate that did not result in a competitive advantage for *Ae. albopictus* larvae (Murrell and Juliano 2014).

The spatial correlation range was 0.9 km for *Ae. aegypti* and 1.0 km for *Ae. albopictus*, indicating that observations farther apart than those distances were uncorrelated. This short range supports previous findings that the dispersal of adult *Ae. aegypti* (Harrington et al. 2001) and *Ae. albopictus* (Medeiros et al. 2017) is below 1 km. *Aedes* adults of these species rarely travel more than 1 km from their hatching site before oviposition and death, highlighting the importance of local conditions in determining mosquito populations.

We evaluated the predictive reliability of our models by conducting simulation studies, then comparing the residuals to the observed mosquito population data. Residual analysis revealed that the predictions for *Ae. albopictus* were not as reliable as those for *Ae. aegypti*, with the model generally underpredicting *Ae. albopictus* counts. Although the observed number of zero observations was within the 95% prediction interval in the *Ae. albopictus* model, the observation was at the extreme high end of predictions, which indicates that the model predicted insufficient zeroes. The use of a zero-inflated model, a standard approach for such situations, did not improve the model fit. Because 84.0% of the observations for *Ae. albopictus* were zero, compared with 17.7% for *Ae. aegypti*, we believe that the relative rarity of *Ae. albopictus* in the study area contributed to poorer model fit due to a relative lack of data. We did not use a subset of the *Ae. albopictus* data with fewer zeroes to train the model because we conducted simultaneous trapping for both *Ae. aegypti* and *Ae. albopictus*, and eliminating negative observations of *Ae. albopictus* would have entailed discarding positive *Ae. aegypti* results. Although the mean number of *Ae. albopictus* observed per week was 46 (SD 41), the model predicted less than five mosquitoes trapped per week at all time points. By comparison, the mean number of *Ae. aegypti* observed per week was 753 (SD 505). Although the model overpredicts the number of *Ae. aegypti*, the discrepancy is much lower in relative magnitude than that for *Ae. albopictus*. The predicted population peak for *Ae. albopictus* in the winter matches the observed data, but the peak for *Ae. aegypti* is predicted several weeks before the peak in the observed data. The peak in *Ae. aegypti* predictions coincides with the peak in shortwave irradiance across the Brownsville area (Supp Fig. 10 [online only]), whereas the peak in observed abundance coincides with temperature (Supp Fig. 7 [online only]). This may indicate the presence of an unmeasured variable affecting *Ae. aegypti* abundance that is correlated with both temperature and shortwave irradiance, such as plant biomass (Marcelis 1993, 1994).

*Aedes aegypti* and *Ae. albopictus* are species of concern for spreading vector-borne disease in southern Texas, and our research highlights differences in their habitat preferences. In hot, semiarid climates, temperature may have a negative effect on mosquito abundance, in contrast to the general association of increased temperatures with increased mosquito activity. We found that *Ae. aegypti* was more abundant throughout the year, and especially in areas with lawns, impervious cover, and high human population density. Our observations and the predicted mosquito count maps indicate that the two mosquito species populations peak at different times of the year and suggest that vector control efforts in Brownsville should focus on the prevention of *Ae. aegypti* because this species is far more numerous than *Ae. albopictus* even when the latter is at its yearly peak population. Our prediction maps indicate the parts of the city that should be targeted for control efforts prior to peak times.

Potential sources of bias in our data set include nonrandom trap placement due to land ownership and access requirements, nonrandom temporal sampling, and bias due to traps being deployed at different times of the day due to personnel limitations. In future studies, it will be important to record the time of day that deployment and sampling take place and include these as potential control variables. The lack of information about existing vector control efforts, including larvicide treatment and adulticide spraying, placed an important limitation on our study. Additionally, winter refugia and cryptic habitats play an important role in determining the abundance of *Aedes* mosquitoes, even in areas with mild winters that do not commonly reach freezing temperatures (Tsunoda et al. 2014). Although the inclusion of a spatial random effect can compensate for unmeasured variables such as pesticide treatment or cryptic habitats, the short range of the spatial effect probably did not compensate for this. Further research with more complete mosquito treatment data will improve disease control and prevention in areas where climate or socioeconomic conditions may influence mosquito population dynamics.

## Supplementary Material

tjz132_suppl_Supplementary-Appendix

## References

[CIT0001] Alto B. W., and Juliano S. A.. 2001 Precipitation and temperature effects on populations of *Aedes albopictus* (Diptera: Culicidae): implications for range expansion. J. Med. Entomol. 38: 646–656. 11580037 10.1603/0022-2585-38.5.646 PMC2579929

[CIT0002] Asare E. O., Tompkins A. M., Amekudzi L. K., Ermert V., and Redl R.. 2016 Mosquito breeding site water temperature observations and simulations towards improved vector-borne disease models for Africa. Geospatial Health 11: 67–77. 10.4081/gh.2016.391 27063735

[CIT0003] Barrera R., Amador M., and Clark G. G.. 2006a Ecological factors influencing *Aedes aegypti* (Diptera: Culicidae) productivity in artificial containers in Salinas, Puerto Rico. J. Med. Entomol. 43: 484–492. 16739405 10.1603/0022-2585(2006)43[484:efiaad]2.0.co;2

[CIT0004] Barrera R., Amador M., and Clark G. G.. 2006b Use of the pupal survey technique for measuring *Aedes aegypti* (Diptera: Culicidae) productivity in Puerto Rico. Am. J. Trop. Med. Hyg. 74: 290–302. 16474086

[CIT0005] Becker B., Leisnham P. T., and LaDeau S. L.. 2014 A tale of two city blocks: differences in immature and adult mosquito abundances between socioeconomically different urban blocks in Baltimore (Maryland, USA). Int. J. Environ. Res. Public Health 11: 3256–3270. 24651396 10.3390/ijerph110303256 PMC3987033

[CIT0006] Brady O. J., Johansson M. A., Guerra C. A., Bhatt S., Golding N., Pigott D. M., Delatte H., Grech M. G., Leisnham P. T., Maciel-de-Freitas R., et al 2013 Modelling adult *Aedes aegypti* and *Aedes albopictus* survival at different temperatures in laboratory and field settings. Parasit. Vectors 6: 351. 24330720 10.1186/1756-3305-6-351 PMC3867219

[CIT0007] Braks M. A. H., Honório N., Lounibos L., Lourenço-de-Oliveira R., and Juliano S.. 2004 Interspecific competition between two invasive species of container mosquitoes, *Aedes aegypti* and *Aedes albopictus* (Diptera: Culicidae), in Brazil. Ann. Entomol. Soc. Am. 97: 130–139.

[CIT0008] Britton C., and Dodd J.. 1976 Relationships of photosynthetically active radiation and shortwave irradiance. Agric. Meteorol. 17: 1–7.

[CIT0009] Carrington L. B., Seifert S. N., Willits N. H., Lambrechts L., and Scott T. W.. 2013 Large diurnal temperature fluctuations negatively influence *Aedes aegypti* (Diptera: Culicidae) life-history traits. J. Med. Entomol. 50: 43–51. 23427651 10.1603/me11242

[CIT0010] Centers for Disease Control and Prevention (CDC) 2007 Dengue hemorrhagic fever – US.-Mexico border, 2005. MMWR Morb. Mortal. Wkly. Rep. 56: 785–789. 17687243

[CIT0011] Centers for Disease Control and Prevention (CDC) 2010 Locally acquired dengue – Key West, Florida, 2009–2010. MMWR Morb. Mortal. Wkly. Rep. 59: 577–581. 20489680

[CIT0012] Chadee D. D., Ward R. A., and Novak R. J.. 1998 Natural habitats of *Aedes Aegypti* in the Caribbean – a review. J. Am. Mosq. Control Assoc. 14: 5–11. 9599318

[CIT0013] Chaves L. F., Morrison A. C., Kitron U. D., and Scott T. W.. 2012 Nonlinear impacts of climatic variability on the density-dependent regulation of an insect vector of disease. Global Change Biol. 18: 457–468.

[CIT0014] Chaves L. F., Scott T. W., Morrison A. C., and Takada T.. 2014 Hot temperatures can force delayed mosquito outbreaks via sequential changes in *Aedes aegypti* demographic parameters in autocorrelated environments. Acta Trop. 129: 15–24. 23537497 10.1016/j.actatropica.2013.02.025

[CIT0015] Costa E. A. P. D. A., Santos E. M. D. M., Correia J. C., and Albuquerque C. M. R. D.. 2010 Impact of small variations in temperature and humidity on the reproductive activity and survival of *Aedes aegypti* (Diptera, Culicidae). Rev. Bras. Entomol. 54: 488–493.

[CIT0016] Crescenti G. H 1997 Meteorological measurements during the Lower Rio Grande Valley environmental study. Environ. Int. 23: 629–642.

[CIT0017] Darsie R. F., and Ward R. A.. 2005 Identification and geographical distribution of the mosquitoes of North America, North of Mexico, 2nd ed University Press of Florida, Gainesville, FL.

[CIT0018] Delatte H., Desvars A., Bouétard A., Bord S., Gimonneau G., Vourc’h G., and Fontenille D.. 2010 Blood-feeding behavior of *Aedes albopictus*, a vector of Chikungunya on La Réunion. Vector Borne Zoonotic Dis. 10: 249–258. 19589060 10.1089/vbz.2009.0026

[CIT0019] Dowling Z., Armbruster P., LaDeau S. L., DeCotiis M., Mottley J., and Leisnham P. T.. 2013 Linking mosquito infestation to resident socioeconomic status, knowledge, and source reduction practices in suburban Washington, DC. Ecohealth 10: 36–47. 23377982 10.1007/s10393-013-0818-6

[CIT0020] Dzul-Manzanilla F., Ibarra-López J., Bibiano Marín W., Martini-Jaimes A., Leyva J. T., Correa-Morales F., Huerta H., Manrique-Saide P., and Vazquez-Prokopec G. M.. 2016 Indoor resting behavior of *Aedes aegypti* (Diptera: Culicidae) in Acapulco, Mexico. J. Med. Entomol. 54: 501–504. 10.1093/jme/tjw203 28011725

[CIT0021] Effler P. V., Pang L., Kitsutani P., Vorndam V., Nakata M., Ayers T., Elm J., Tom T., Reiter P., Rigau-Perez J. G., et al.; Hawaii Dengue Outbreak Investigation Team. 2005 Dengue fever, Hawaii, 2001–2002. Emerg. Infect. Dis. 11: 742–749. 15890132 10.3201/eid1105.041063 PMC3320380

[CIT0022] Farjana T., Tuno N., and Higa Y.. 2012 Effects of temperature and diet on development and interspecies competition in *Aedes aegypti* and *Aedes albopictus*. Med. Vet. Entomol. 26: 210–217. 21781139 10.1111/j.1365-2915.2011.00971.x

[CIT0023] Fuglstad G.-A., Simpson D., Lindgren F., and Rue H.. 2018 Constructing priors that penalize the complexity of Gaussian random fields. J. Am. Stat. Assoc. 525: 1–8.

[CIT0024] George E. I., and McCulloch R. E.. 1997 Approaches for Bayesian variable selection. Stat. Sinica 7: 339–373.

[CIT0025] Hamer D. H., and Chen L. H.. 2014 Chikungunya: establishing a new home in the Western hemisphere. Ann. Intern. Med. 161: 827–828. 25244354 10.7326/M14-1958

[CIT0026] Harrington L. C., Buonaccorsi J. P., Edman J. D., Costero A., Kittayapong P., Clark G. G., and Scott T. W.. 2001 Analysis of survival of young and old *Aedes aegypti* (Diptera: Culicidae) from Puerto Rico and Thailand. J. Med. Entomol. 38: 537–547. 11476334 10.1603/0022-2585-38.4.537

[CIT0027] Hawley W. A., Reiter P., Copeland R. S., Pumpuni C. B., and Craig G. B. Jr 1987 *Aedes albopictus* in North America: probable introduction in used tires from northern Asia. Science 236: 1114–1116. 3576225 10.1126/science.3576225

[CIT0028] Ho B. C., Ewert A., and Chew L. M.. 1989 Interspecific competition among *Aedes aegypti*, *Ae. albopictus*, and *Ae. triseriatus* (Diptera: Culicidae): larval development in mixed cultures. J. Med. Entomol. 26: 615–623. 2585456 10.1093/jmedent/26.6.615

[CIT0029] Hylton A. R 1967 Low humidity water-retention ability in *Eretmapodites chrysogaster* and *Aedes albopictus*. J. Insect Physiol. 13: 153–157. 4382866 10.1016/0022-1910(67)90011-x

[CIT0030] Jacobs A., Heusinkveld B., and Nieveen J.. 1998 Temperature behavior of a natural shallow water body during a summer period. Theor. Appl. Climatol. 59: 121–127.

[CIT0031] Juliano S. A., O’Meara G. F., Morrill J. R., and Cutwa M. M.. 2002 Desiccation and thermal tolerance of eggs and the coexistence of competing mosquitoes. Oecologia 130: 458–469. 20871747 10.1007/s004420100811 PMC2944657

[CIT0032] Kraemer M. U. G., Reiner R. C. Jr, Brady O. J., Messina J. P., Gilbert M., Pigott D. M., Yi D., Johnson K., Earl L., Marczak L. B., et al 2019 Past and future spread of the arbovirus vectors *Aedes aegypti* and *Aedes albopictus*. Nat. Microbiol. 4: 901. 30962571 10.1038/s41564-019-0440-7 PMC7609323

[CIT0033] Laird N. M., and Ware J. H.. 1982 Random-effects models for longitudinal data. Biometrics 38: 963–974. 7168798

[CIT0034] Landau K. I., and van Leeuwen W. J.. 2012 Fine scale spatial urban land cover factors associated with adult mosquito abundance and risk in Tucson, Arizona. J. Vector Ecol. 37: 407–418. 23181866 10.1111/j.1948-7134.2012.00245.x

[CIT0035] Lindgren F., Rue H., and Lindstrom J.. 2011 An explicit link between Gaussian fields and Gaussian Markov random fields: the SPDE approach (with discussion). J. R. Stat. Soc. B 73: 423–498.

[CIT0036] Lounibos L. P., Suárez S., Menéndez Z., Nishimura N., Escher R. L., O’Connell S. M., and Rey J. R.. 2002 Does temperature affect the outcome of larval competition between *Aedes aegypti* and *Aedes albopictus*? J. Vector Ecol. 27: 86–95. 12125878

[CIT0037] Marcelis L 1993 Fruit growth and biomass allocation to the fruits in cucumber. 2. Effect of irradiance. Sci. Horticult. 54: 123–130.

[CIT0038] Marcelis L 1994 Effect of fruit growth, temperature and irradiance on biomass allocation to the vegetative parts of cucumber. NJAS Wageningen J. Life Sci. 42: 115–123.

[CIT0039] Medeiros M. C., Boothe E. C., Roark E. B., and Hamer G. L.. 2017 Dispersal of male and female *Culex quinquefasciatus* and *Aedes albopictus* mosquitoes using stable isotope enrichment. PLoS Negl. Trop. Dis. 11: e0005347. 28135281 10.1371/journal.pntd.0005347 PMC5300284

[CIT0040] Monaghan A. J., Morin C. W., Steinhoff D. F., Wilhelmi O., Hayden M., Quattrochi D. A., Reiskind M., Lloyd A. L., Smith K., Schmidt C. A., et al 2016 On the seasonal occurrence and abundance of the Zika virus vector mosquito *Aedes aegypti* in the contiguous United States. PLoS Currents 8. 10.1371/currents.outbreaks.50dfc7f46798675fc63e7d7da563da76 PMC4807952 27066299

[CIT0041] Muir L. E., and Kay B. H.. 1998 *Aedes aegypti* survival and dispersal estimated by mark-release-recapture in northern Australia. Am. J. Trop. Med. Hyg. 58: 277–282. 9546403 10.4269/ajtmh.1998.58.277

[CIT0042] Murrell E. G., and Juliano S. A.. 2014 Detritus type alters the outcome of interspecific competition between *Aedes aegypti* and *Aedes albopictus* (Diptera: Culicidae). J. Med. Entomol. 45: 375–383. 10.1603/0022-2585(2008)45[375:dtatoo]2.0.co;2 PMC2583230 18533429

[CIT0043] Pan American Health Organization 2017 Countries and territories with autochthonous transmission of Zika virus in the Americas reported in 2015–2017. Regional Office for the Americas of the World Health Organization, Washington, D.C.

[CIT0044] Peel M. C., Finlayson B. L., and McMahon T. A.. 2007 Updated world map of the Köppen-Geiger climate classification. Hydrol. Earth Syst. Sci. Discuss. 4: 439–473.

[CIT0045] Perich M. J., Davila G., Turner A., Garcia A., and Nelson M.. 2000 Behavior of resting *Aedes aegypti* (Culicidae: Diptera) and its relation to ultra-low volume adulticide efficacy in Panama City, Panama. J. Med. Entomol. 37: 541–546. 10916294 10.1603/0022-2585-37.4.541

[CIT0046] R Core Team 2018 R: a language and environment for statistical computing. R Foundation for Statistical Computing, Vienna, Austria.

[CIT0047] Rey J. R., Nishimura N., Wagner B., Braks M. A., O’Connell S. M., and Lounibos L. P.. 2006 Habitat segregation of mosquito arbovirus vectors in south Florida. J. Med. Entomol. 43: 1134–1141. 17162945 10.1603/0022-2585(2006)43[1134:hsomav]2.0.co;2 PMC1820833

[CIT0048] Rue H., Martino S., and Chopin N.. 2009 Approximate Bayesian inference for latent Gaussian models using integrated nested Laplace approximations (with discussion). J. R. Stat. Soc. B 71: 319–392.

[CIT0049] Ruiz M. O., Tedesco C., McTighe T. J., Austin C., and Kitron U.. 2004 Environmental and social determinants of human risk during a West Nile virus outbreak in the greater Chicago area, 2002. Int. J. Health Geogr. 3: 8. 15099399 10.1186/1476-072X-3-8 PMC420251

[CIT0050] Sallam M. F., Fizer C., Pilant A. N., and Whung P.-Y.. 2017 Systematic review: land cover, meteorological, and socioeconomic determinants of *Aedes* mosquito habitat for risk mapping. Int. J. Environ. Res. Public Health 14: 1230. 10.3390/ijerph14101230 PMC5664731 29035317

[CIT0051] Samson D. M., Qualls W. A., Roque D., Naranjo D. P., Alimi T., Arheart K. L., Müller G. C., Beier J. C., and Xue R. D.. 2013 Resting and energy reserves of *Aedes albopictus* collected in common landscaping vegetation in St. Augustine, Florida. J. Am. Mosq. Control Assoc. 29: 231–236. 24199497 10.2987/13-6347R.1 PMC3921969

[CIT0052] Schiepl F., Fahrmeir L., and Kneib T.. 2012 Spike-and-slab priors for function selection in structured additive regression models. J. Am. Stat. Assoc. 107: 1518–1532.

[CIT0053] Sprenger P. R. D 1987 The used tire trade: a mechanism for the worldwide dispersal of container breeding mosquitoes. J. Am. Mosq. Control. Assoc. 3: 494. 2904963

[CIT0054] Sprenger D., and Wuithiranyagool T.. 1986 The discovery and distribution of *Aedes albopictus* in Harris County, Texas. J. Am. Mosq. Control Assoc. 2: 217–219. 3507493

[CIT0055] Thornton P. E., Thornton M. M., Mayer B. W., Wei Y., Devarakonda R., Vose R. S., and Cook R. B.. 2016 Daymet: daily surface weather data on a 1-km grid for North America, version 3. ORNL DAAC, Oak Ridge, TN.

[CIT0056] Tsunoda T., Cuong T. C., Dong T. D., Yen N. T., Le N. H., Phong T. V., and Minakawa N.. 2014 Winter refuge for *Aedes aegypti* and *Ae. albopictus* mosquitoes in Hanoi during Winter. PLoS One 9: e95606. 24752230 10.1371/journal.pone.0095606 PMC3994068

[CIT0057] U.S. Census Bureau 2018 2013–2017 American Community Survey 5-year estimates. American Community Survey. Retrieved from: https://www.census.gov/programs-surveys/acs/technical-documentation/table-and-geography-changes/2017/5-year.html.

[CIT0058] Yang H. M., Macoris M. L., Galvani K. C., Andrighetti M. T., and Wanderley D. M.. 2009 Assessing the effects of temperature on the population of *Aedes aegypti*, the vector of dengue. Epidemiol. Infect. 137: 1188–1202. 19192322 10.1017/S0950268809002040

