# Supplementary material for: Mapping Aedes aegypti (Diptera: Culicidae) and Aedes albopictus Vector Mosquito Distribution in Brownsville, TX
Source: J Med Entomol. 2019 Aug 10;57(1):231–40. doi: 10.1093/jme/tjz132 (PMC6951034; doi:10.1093/jme/tjz132)
Supplement: tjz132_suppl_Supplementary-Appendix [file tjz132_suppl_supplementary-appendix.docx]

# Technical Appendix

# Mapping *Aedes aegypti* and *Ae. albopictus* vector mosquito distribution in Brownsville, Texas

Mark H. Myer, Chelsea Fizer, Kenneth McPherson, Anne Neale, Andrew Pilant, Arturo Rodriguez, Pai-Yei Whung, and John M. Johnston*

Author Affiliations: U.S. Environmental Protection Agency, Athens, Georgia, USA (M.H. Myer, J.M. Johnston, C. Fizer, A. Pilant, A. Neale, P-Y Whung); Public Health Department, Brownsville, Texas, USA (A. Rodriguez); and U.S. Environmental Protection Agency, Dallas, Texas, USA (K. McPherson).

*Corresponding Author. Mail: 960 College Station Road, Athens, Georgia, 30605, USA. E-mail: [johnston.johnm@epa.gov](mailto:johnston.johnm@epa.gov). Telephone: (706) 855-8300

## Mosquito sampling

To determine whether there was temporal bias in sampling, we plotted the number of individual trap samples taken per week **(Appendix Figure 1)** and the samples taken per trap **(Appendix Figure 2)**. The mean number of trap samples taken per week was 114, and the mean number of samples taken per trap was 67. We chose a model, hierarchical Bayesian mixed effects modeling, that is robust to unbalanced sampling to compensate for the lack of evenness in temporal and spatial sampling.


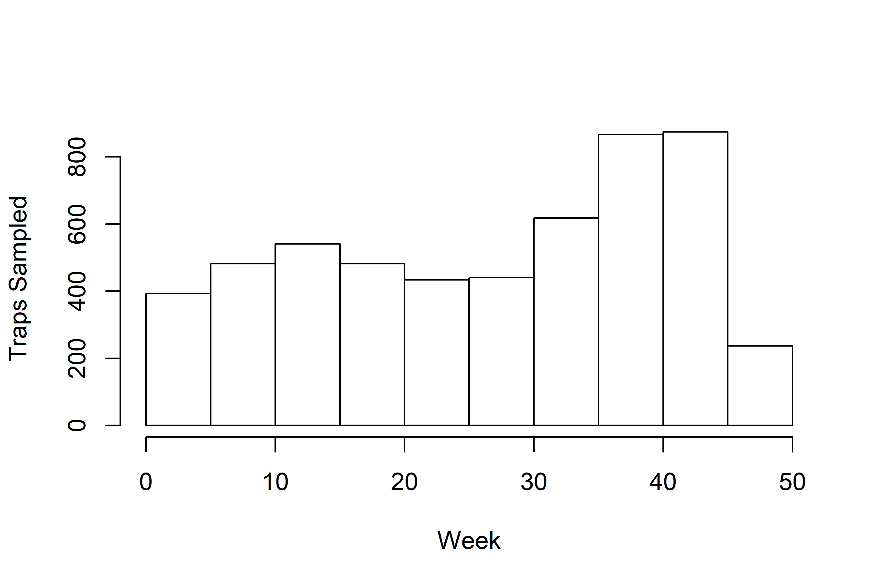


**Appendix Figure 1.** Mosquito trap samples per ordinal week in 2017. Trap sampling effort was the highest the week of October 23^rd^, and lowest the week of January 1^st^.


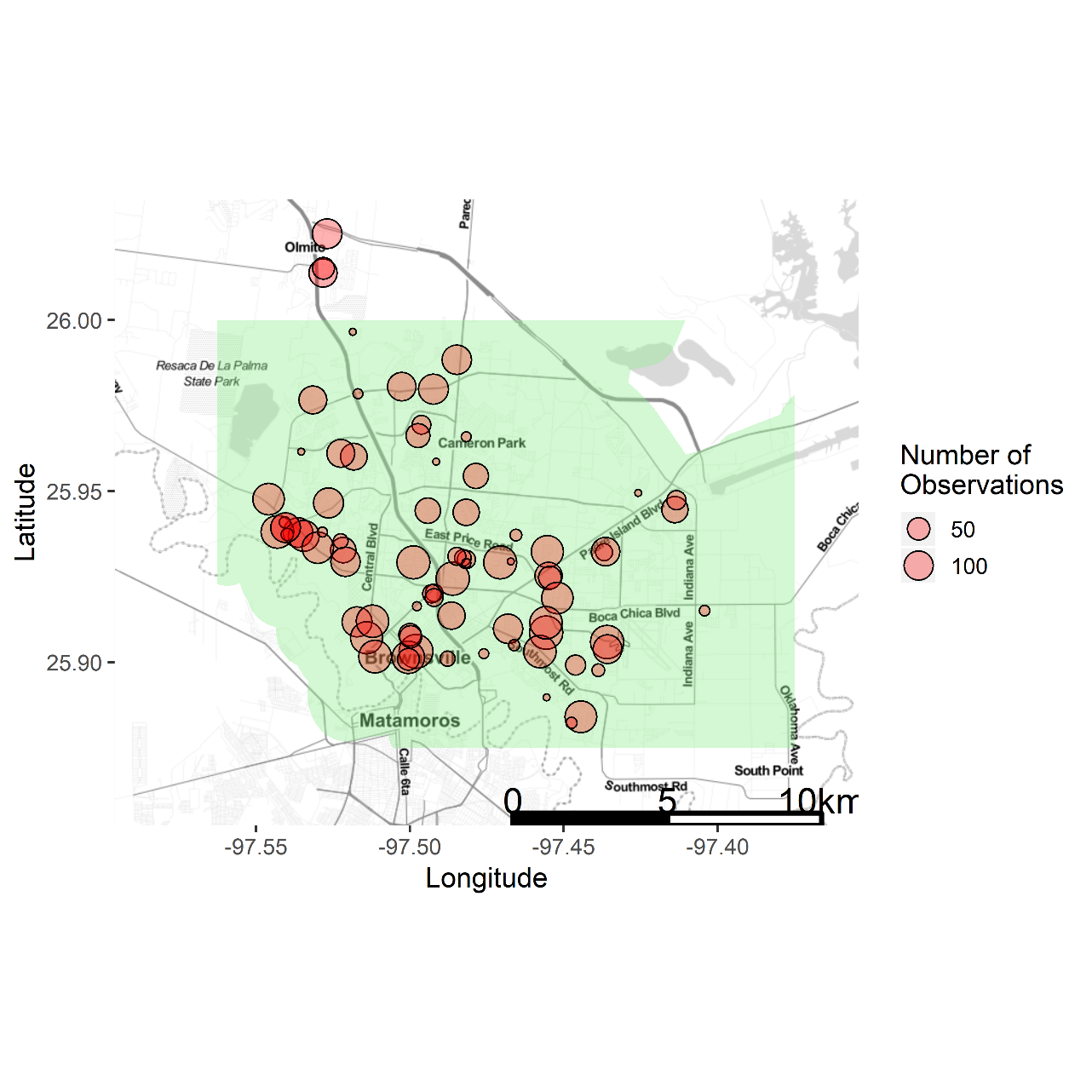


**Appendix Figure 2.** Mosquito trap locations sized by the number of observations taken in 2017. Smaller circles correspond to fewer observations.

We eliminated trap sites that had fewer than 30 observations due to concerns about their sampling frequency **(Appendix Figure 3)**. The removed traps had a total of 288 observations, representing 5.4% of total mosquito trap samples. The mean number of samples taken per trap after removing the trap sites with few observations was 100. We did not note any pattern or clustering in the traps that were eliminated from consideration.


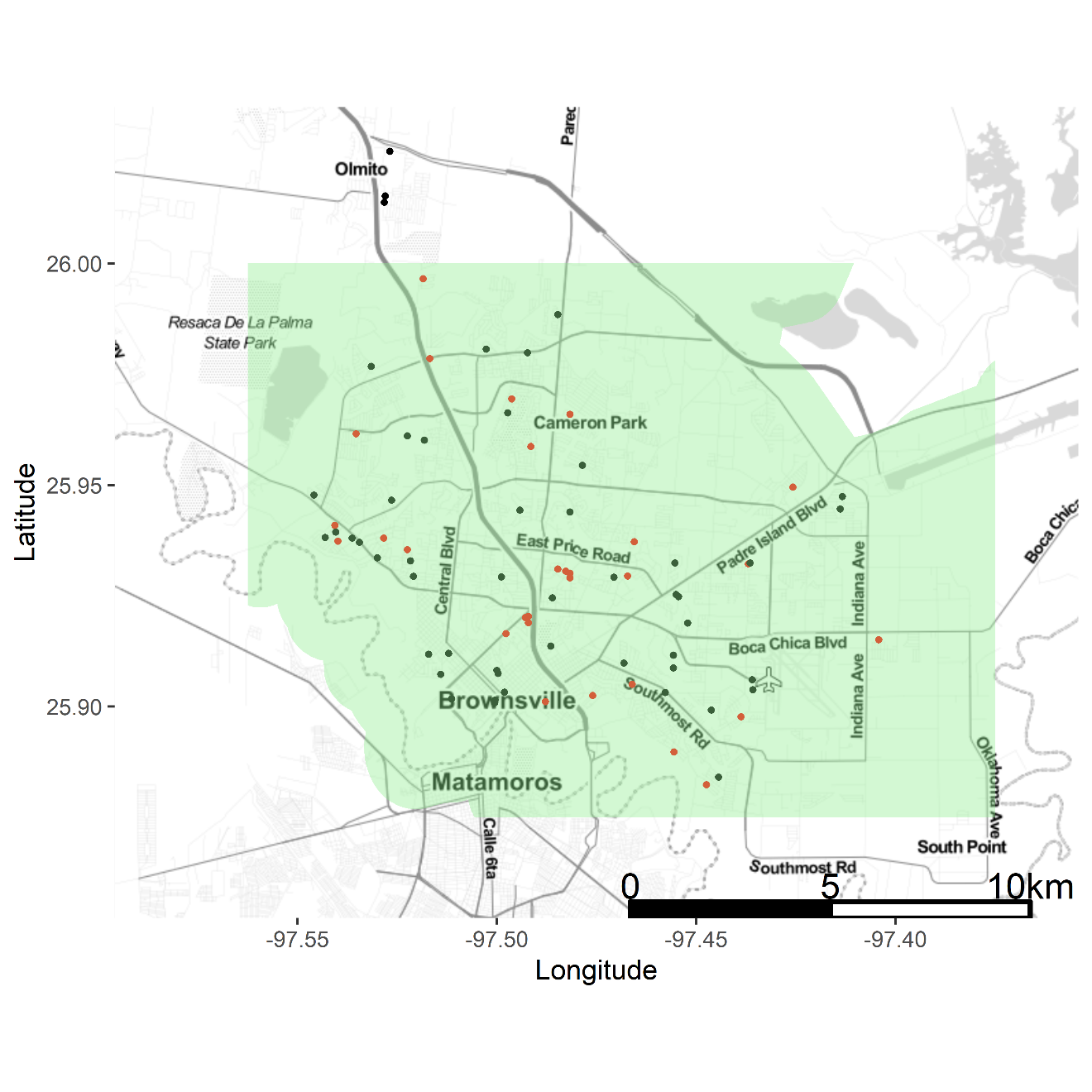


**Appendix Figure 3.** Locations of BT-Sentinel traps with removed traps (< 30 observations) highlighted in red (n = 29).

To illustrate the placement of traps in context with land cover, we plotted traps overlaid on 1m land cover for Brownsville in **Appendix Figure 4.**


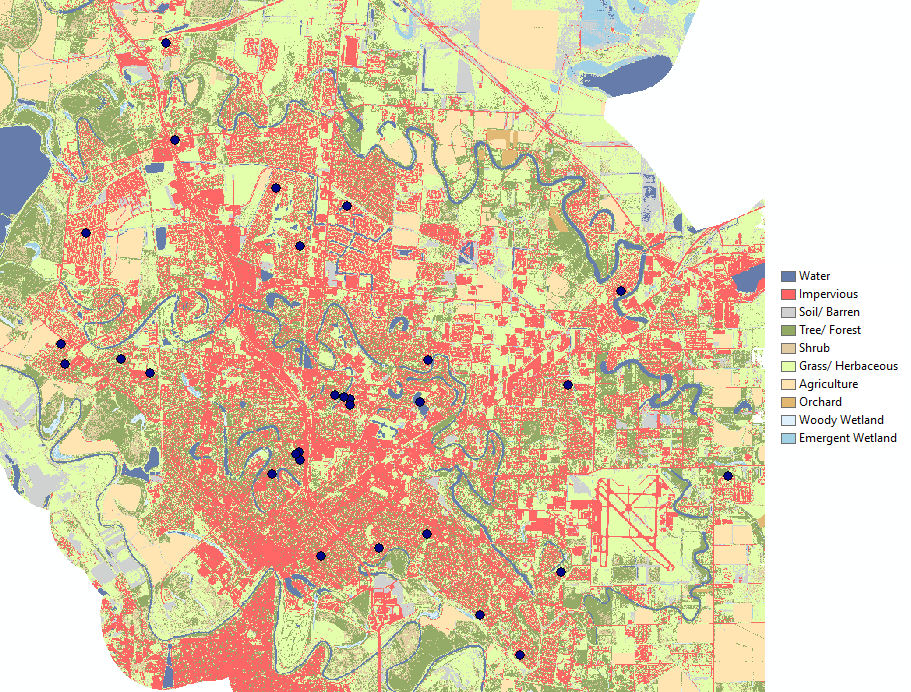


**Appendix Figure 4.** Locations of traps overlaid on 1m resolution land cover for Brownsville, TX.

## Correlation plots and correlation coefficients for selected variables

We created scatterplots to help visualize correlations in the variables chosen to model counts of each mosquito species **(Appendix Figure 4)**.


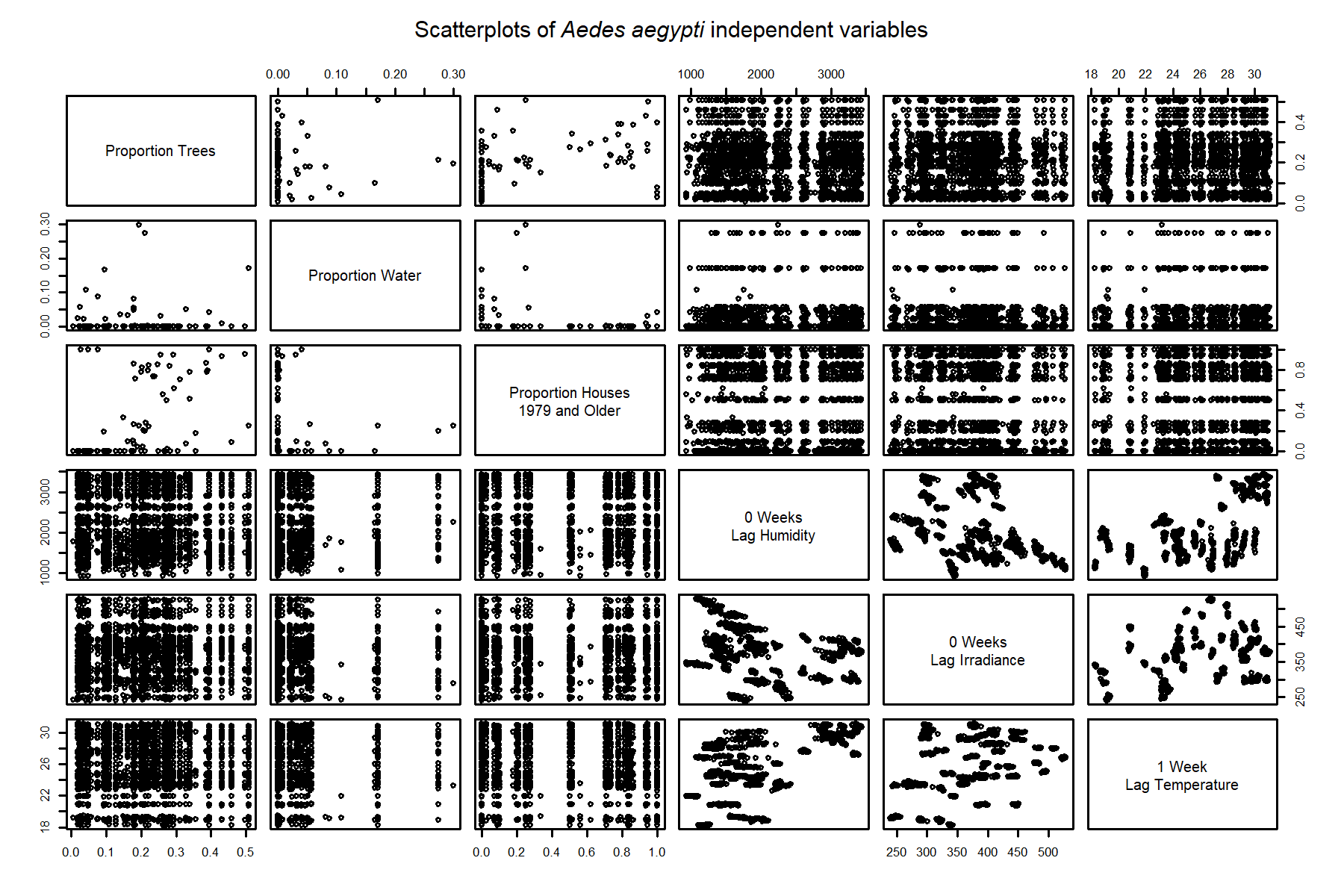


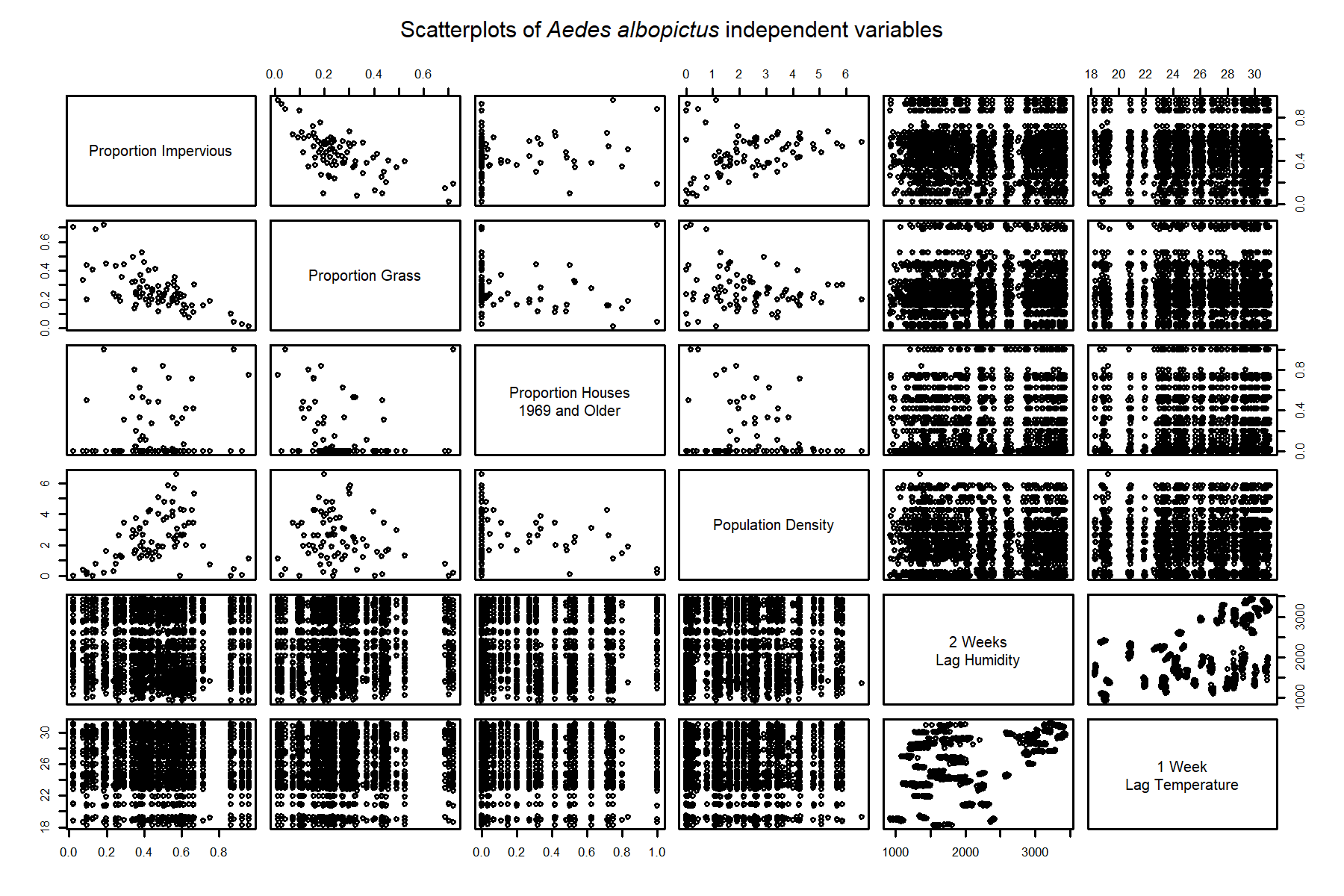


**Appendix Figure 5.** Scatterplots for selected independent variables for *Aedes aegypti* and *Aedes albopictus*.

**Appendix Table 1**. Spearman’s correlation coefficients for selected independent variables for *Aedes aegypti*

|  | Proportion Trees | Proportion Water | Proportion Houses 1979 and Older | 0 Weeks Lag Humidity | 0 Weeks Lag Irradiance | 1 Weeks Lag Temperature |
| --- | --- | --- | --- | --- | --- | --- |
| Proportion Trees | 1 | 0.11 | 0.41 | -0.01 | 0 | -0.02 |
| Proportion Water | 0.11 | 1 | 0.10 | -0.04 | -0.02 | -0.06 |
| Proportion Houses 1979 and Older | 0.41 | 0.10 | 1 | -0.11 | 0.01 | 0.01 |
| 0 Weeks Lag Humidity | -0.01 | -0.04 | -0.11 | 1 | -0.33 | 0.61 |
| 0 Weeks Lag Irradiance | 0 | -0.02 | 0.01 | -0.33 | 1 | 0.25 |
| 1 Weeks Lag Temperature | -0.02 | -0.06 | 0.01 | 0.61 |  | 1 |

**Appendix Table 2**. Spearman’s correlation coefficients for selected independent variables for *Aedes albopictus*

|  | Proportion Impervious | Proportion Grass | Proportion Houses 1969 and Older | Population Density | 2 Weeks Lag Humidity | 1 Weeks Lag Temperature |
| --- | --- | --- | --- | --- | --- | --- |
| Proportion Impervious | 1 | -0.67 | -0.04 | 0.22 | -0.02 | 0 |
| Proportion Grass | -0.67 | 1 | -0.11 | -0.12 | 0.02 | 0.01 |
| Proportion Houses 1969 and Older | -0.04 | -0.11 | 1 | 0.13 | 0 | 0.02 |
| Population Density | 0.22 | -0.12 | 0.13 | 1 | 0.01 | 0.01 |
| 2 Weeks Lag Humidity | -0.02 | 0.02 | 0 | 0.01 | 1 | 0.54 |
| 1 Weeks Lag Temperature | 0 | 0.01 | 0.02 | 0.01 |  | 1 |

## Interaction testing for correlated variables

To determine whether correlated variables affected interpretation of the negative binomial spatial INLA models, we removed one of each pair and compared the resulting variable coefficients, then we checked an interaction term to see if it improved model fit or the interpretation of the model.

### Aedes aegypti

This model contained one pair of correlated variables: Temperature and Humidity (ρ = 0.61). The table of model results with the full set of variables and no interactions is listed in **Appendix Table 2** for reference:

**Appendix Table 2.** Variable coefficients for Aedes aegypti negative binomial spatial model with all variables selected by Bayesian variable selection

|  | | **Coefficient (RR^1^)** | |
| --- | --- | --- | --- |
| **Variable** | | **Mean** | **95% CI^2^** |
| Intercept | | 1.53 | 1.28-1.78 |
| Trees | | 0.34 | 0.15-0.53 |
| Water | | -0.01 | -0.15 to 0.12 |
| Built before 1979 | | 0.09 | -0.04-0.22 |
| Humidity | | 0.46 | 0.41-0.51 |
| Irradiance | | 0.44 | 0.40-0.48 |
| Lag 1 Temperature | | -0.17 | -0.22 to -0.13 |
| **Parameter** | |  |  |
| Dispersion | | 0.92 | 0.72-0.97 |
| Spatial range | | 0.9 km | 0.5-1.5 km |
| **Indicator of Fit** |  | |  |
| DIC^3^ | 29065.29 | |  |

^1^Relative risk

^2^Credible interval

^3^Deviance information criterion

Leaving out Humidity **(Appendix Table 3)** resulted in the coefficient of Lag 1 Temperature reversing in sign, while the 95% CI did not encompass zero and the variable retained its statistical importance. Humidity was acting as a suppressor variable towards Lag 1 Temperature in this model. We interpreted this result to mean that at a constant, controlled Humidity, Aedes aegypti trap counts are lowered with increasing temperature. The general trend, however, is for increased temperature to result in increased Aedes aegypti counts, a finding that is supported extensively in entomological literature. The other variables were unchanged in interpretation, though the coefficient of Irradiance was lowered. All weather variables are correlated to some degree, so change in the coefficient for Irradiance was expected. The DIC was higher than the full model, suggesting that the model with both Temperature and Humidity included was a better fit for the data despite multicollinearity. Because the correlation coefficient was not precisely 1 or -1, there is information contained in one variable that is not present in the other, and the model is more informative with both included. Multicollinearity does not impair model fit or predictions. It is important, however, to carefully consider the interpretation of coefficients of correlated variables as we have done here.

**Appendix Table 3.** Variable coefficients for Aedes aegypti negative binomial spatial model with Humidity dropped

|  | **Coefficient (RR)** | |
| --- | --- | --- |
| **Variable** | **Mean** | **95% CI** |
| Intercept | 1.53 | 1.26-1.79 |
| Trees | 0.32 | 0.13-0.51 |
| Water | -0.02 | -0.15 to 0.12 |
| Built before 1979 | 0.10 | -0.02-0.23 |
| Irradiance | 0.21 | 0.18-0.24 |
| Lag 1 Temperature | 0.17 | 0.13-0.20 |
| **Parameter** |  |  |
| Dispersion | 1.0 | 0.95-1.05 |
| Spatial range | 1 km | 0.6-1.7 km |
| **Indicator of Fit** |  |  |
| DIC | 29388.29 |  |

Leaving out Lag 1 Temperature **(Appendix Table 4)** resulted in no changes in the interpretation of the model compared to the full initial model with all 6 chosen variables retained, although as before the two weather variables changed in magnitude due to their correlation with temperature.

**Appendix Table 4.** Variable coefficients for Aedes aegypti negative binomial spatial model with Lag 1 Temperature dropped

|  | **Coefficient (RR)** | |
| --- | --- | --- |
| **Variable** | **Mean** | **95% CI** |
| Intercept | 1.54 | 1.28-1.79 |
| Trees | 0.34 | 0.15-0.54 |
| Water | -0.02 | -0.15 to 0.12 |
| Built before 1979 | 0.09 | -0.04-0.22 |
| Irradiance | 0.35 | 0.32-0.39 |
| Humidity | 0.32 | 0.29-0.36 |
| **Parameter** |  |  |
| Dispersion | 0.93 | 0.89-0.98 |
| Spatial range | 0.9 km | 0.7-1.6 km |
| **Indicator of Fit** |  |  |
| DIC | 29114.02 |  |

Including an interaction term for Lag 1 Temperature x Humidity **(Appendix Table 5)** resulted in a negative and important coefficient, and a moderate decrease in DIC indicating marginally improved model fit. It is important to note that because our variables are scaled by subtracting the mean and dividing by the standard deviation, they are centered at zero. The interpretation of a main effect in a model with an interaction term is “the effect of a 1 unit increase in this variable when the other in the interaction is zero”. For this model, that means that the interpretation of the main effects for Humidity and Lag 1 Temperature are conditional upon the other variable being at its mean value. We found that at mean Humidity, Lag 1 Temperature remains negative and important. Similarly, at mean Lag 1 Temperature, Humidity remains positive and important. This confirms our finding previously that at a given level of humidity, increased temperature results in fewer *Aedes aegypti* counted in our sample. The negative interaction term confirms that a higher value of Humidity results in a more negative effect of Temperature, and vice versa.

**Appendix Table 5**. Variable coefficients for Aedes aegypti negative binomial spatial model with interaction term between Lag 1 Temperature and Humidity

|  | | **Coefficient (RR)** | | |
| --- | --- | --- | --- | --- |
| **Variable** | | **Mean** | **95% CI** | |
| Intercept | | 1.59 | | 1.33-1.83 |
| Trees | | 0.34 | | 0.15-0.53 |
| Water | | -0.01 | | -0.15 to 0.12 |
| Built before 1979 | | 0.09 | | -0.04-0.22 |
| Humidity | | 0.46 | | 0.41-0.51 |
| Irradiance | | 0.52 | | 0.46-0.57 |
| Lag 1 Temperature | | -0.21 | | -0.26 to -0.16 |
| Humidity x Lag 1 Temperature | | -0.08 | | -0.12 to -0.04 |
| **Parameter** | |  |  | |
| Dispersion | | 0.92 | 0.72-0.97 | |
| Spatial range | | 0.9 km | 0.5-1.5 km | |
| **Indicator of Fit** |  | |  | |
| DIC | 29053.57 | |  | |

### Aedes albopictus

This model contained two pairs of correlated variables: Temperature and Humidity (ρ = 0.61); and Impervious and Grass (ρ = -0.67). The table of model results with the full set of variables and no interactions is listed in **Appendix Table 6** for reference:

**Appendix Table 6.** Variable coefficients for Aedes albopictus negative binomial spatial model with all variables selected by Bayesian variable selection

|  | | **Coefficient (RR)** | |
| --- | --- | --- | --- |
| **Variable** | | **Mean** | **95% CI** |
| Intercept | | -1.80 | -2.22 to -1.40 |
| Impervious | | -0.67 | -1.03 to -0.30 |
| Grass | | -0.61 | -0.91 to -0.32 |
| Built before 1969 | | -0.01 | -0.19-0.17 |
| Population Density | | -0.61 | -0.95 to -0.27 |
| Lag 2 Humidity | | 0.51 | 0.41-0.61 |
| Lag 1 Temperature | | -0.49 | -0.58 to -0.39 |
| **Parameter** | |  |  |
| Dispersion | | 2.29 | 2.01-2.63 |
| Spatial range | | 1.4 km | 1.1-1.7 km |
| **Indicator of Fit** |  | |  |
| DIC | 6314.11 | |  |

Leaving out Lag 2 Humidity **(Appendix Table 7)** resulted in Lag 1 Temperature remaining negative and important, in contrast to the result for *Aedes aegypti*. This means that, controlling for the other variables, increasing temperature was associated with reduced counts of *Aedes albopictus* in our sample. This result agrees with the observed dynamics of the two species in our study area throughout the year, with *Aedes aegypti* relatively more numerous in the summer and *Aedes albopictus* experiencing peaks in trap counts in the cooler months (Main manuscript, Fig 2).

**Appendix Table 7.** Variable coefficients for Aedes albopictus negative binomial spatial model with Lag 2 Humidity dropped

|  | | **Coefficient (RR)** | |
| --- | --- | --- | --- |
| **Variable** | | **Mean** | **95% CI** |
| Intercept | | -1.73 | -2.22 to -1.40 |
| Impervious | | -0.78 | -1.16 to -0.41 |
| Grass | | -0.69 | -1.0 to -0.40 |
| Built before 1969 | | 0.04 | -0.14-022 |
| Population Density | | -0.61 | -0.95 to -0.26 |
| Lag 1 Temperature | | -0.22 | -0.29 to -0.14 |
| **Parameter** | |  |  |
| Dispersion | | 2.56 | 2.27-2.2.94 |
| Spatial range | | 1.4 km | 1.1-1.8 km |
| **Indicator of Fit** |  | |  |
| DIC | 6411.01 | |  |

Leaving out Lag 1 Temperature **(Appendix Table 8)** did not result in a change to the interpretation of any variable coefficients.

**Appendix Table 8.** Variable coefficients for Aedes albopictus negative binomial spatial model with Lag 1 Temperature dropped

|  | | **Coefficient (RR)** | |
| --- | --- | --- | --- |
| **Variable** | | **Mean** | **95% CI** |
| Intercept | | -1.67 | -2.09 to -1.27 |
| Impervious | | -0.67 | -1.05 to -0.31 |
| Grass | | -0.67 | -0.97 to -0.37 |
| Built before 1969 | | -0.01 | -0.19-0.17 |
| Population Density | | -0.61 | -0.95 to -0.26 |
| Lag 2 Humidity | | 0.22 | 0.13-0.30 |
| **Parameter** | |  |  |
| Dispersion | | 2.56 | 2.27-2.94 |
| Spatial range | | 1.4 km | 1.1-1.7 km |
| **Indicator of Fit** |  | |  |
| DIC | 6416.57 | |  |

Including an interaction term between the Lag 1 Temperature and Lag 2 Humidity **(Appendix Table 9)** did not result in a large change to any variable coefficients and resulted in a minimal improvement in DIC. The interaction term was negative and important, indicating that at higher humidity, there was a correspondingly more negative effect of temperature and vice versa.

**Appendix Table 9**. Variable coefficients for Aedes albopictus negative binomial spatial model with interaction term between Lag 1 Temperature and Lag 2 Humidity

|  | | **Coefficient (RR)** | |
| --- | --- | --- | --- |
| **Variable** | | **Mean** | **95% CI** |
| Intercept | | -1.72 | -2.14 to -1.31 |
| Impervious | | -0.64 | -1.01 to -0.28 |
| Grass | | -0.61 | -0.91 to -0.32 |
| Built before 1969 | | -0.02 | -0.20-0.16 |
| Population Density | | -0.62 | -0.96 to -0.28 |
| Lag 2 Humidity | | 0.55 | 0.44-0.65 |
| Lag 1 Temperature | | -0.50 | -0.59 to -0.41 |
| Lag 1 Temperature x Lag 2 Humidity | | -0.13 | -0.23 to -0.04 |
| **Parameter** | |  |  |
| Dispersion | | 2.27 | 2.00-2.63 |
| Spatial range | | 1.4 km | 1.1-1.7 km |
| **Indicator of Fit** |  | |  |
| DIC | 6308.75 | |  |

Leaving out Impervious **(Appendix Table 10)** did not result in a change to the interpretation of any variable coefficients. The coefficient of Grass was smaller in magnitude, indicating that Grass and Impervious may have a positive interaction effect.

**Appendix Table 10.** Variable coefficients for Aedes albopictus negative binomial spatial model with Impervious dropped

|  | | **Coefficient (RR)** | |
| --- | --- | --- | --- |
| **Variable** | | **Mean** | **95% CI** |
| Intercept | | -1.78 | -2.24 to -1.35 |
| Grass | | -0.40 | -0.68 to -0.13 |
| Built before 1969 | | -0.03 | -0.21-0.15 |
| Population Density | | -0.84 | -1.18 to -0.50 |
| Lag 2 Humidity | | 0.52 | 0.42-0.62 |
| Lag 1 Temperature | | -0.49 | -0.58 to -0.39 |
| **Parameter** | |  |  |
| Dispersion | | 2.33 | 2.04-2.63 |
| Spatial range | | 1.5 km | 1.2-1.8 km |
| **Indicator of Fit** |  | |  |
| DIC | 6319.38 | |  |

Leaving out Grass **(Appendix Table 11)** did not result in a change to the interpretation of any variable coefficients. The coefficient of Impervious was smaller, indicating that there may be a positive interaction effect.

**Appendix Table 11.** Variable coefficients for Aedes albopictus negative binomial spatial model with Grass dropped

|  | | **Coefficient (RR)** | |
| --- | --- | --- | --- |
| **Variable** | | **Mean** | **95% CI** |
| Intercept | | -1.93 | -2.38 to -1.52 |
| Impervious | | -0.37 | -0.74 to -0.02 |
| Built before 1969 | | -0.10 | -0.28-0.08 |
| Population Density | | -0.68 | -1.05 to -0.32 |
| Lag 2 Humidity | | 0.52 | 0.42-0.62 |
| Lag 1 Temperature | | -0.49 | -0.58 to -0.40 |
| **Parameter** | |  |  |
| Dispersion | | 2.33 | 2.04-2.63 |
| Spatial range | | 1.5 km | 1.2-1.8 km |
| **Indicator of Fit** |  | |  |
| DIC | 6318.89 | |  |

Including an interaction term between Grass and Impervious **(Appendix Table 12)** resulted in a reduction of the coefficients for both Impervious and Grass relative to the model without interactions, and the coefficent for Grass was no longer important. The interaction term was positive and important, indicating that as expected Grass and Impervious have a positive interaction effect. The negative effect of impervious cover on expected counts of *Aedes albopictus* is higher in magnitude when there is less grass.

**Appendix Table 12**. Variable coefficients for Aedes albopictus negative binomial spatial model with interaction term between Grass and Impervious

|  | | **Coefficient (RR)** | |
| --- | --- | --- | --- |
| **Variable** | | **Mean** | **95% CI** |
| Intercept | | -1.58 | -2.05 to -1.12 |
| Impervious | | -0.43 | -0.83 to -0.04 |
| Grass | | -0.26 | -0.65 to -0.14 |
| Built before 1969 | | 0.01 | -0.17-0.19 |
| Population Density | | -0.78 | -1.14 to -0.42 |
| Lag 2 Humidity | | 0.51 | 0.41-0.61 |
| Lag 1 Temperature | | -0.49 | -0.58 to -0.39 |
| Impervious x Grass | | 0.42 | 0.10-0.75 |
| **Parameter** | |  |  |
| Dispersion | | 2.27 | 2.00-2.63 |
| Spatial range | | 1.4 km | 1.1-1.7 km |
| **Indicator of Fit** |  | |  |
| DIC | 6312.12 | |  |

Including both interaction terms **(Appendix Table 13)** resulted in no additional conclusions beyond those stated previously.

**Appendix Table 13.** Variable coefficients for Aedes albopictus negative binomial spatial model with interaction terms between Grass and Impervious and between Lag 1 Temperature and Lag 2 Humidity

|  | | **Coefficient (RR)** | |
| --- | --- | --- | --- |
| **Variable** | | **Mean** | **95% CI** |
| Intercept | | -1.50 | -1.97 to -1.04 |
| Impervious | | -0.41 | -0.80 to -0.02 |
| Grass | | -0.25 | -0.65-0.18 |
| Built before 1969 | | 0.00 | -0.18-0.18 |
| Population Density | | -0.80 | -1.16 to -0.44 |
| Lag 2 Humidity | | 0.55 | 0.44-0.65 |
| Lag 1 Temperature | | -0.50 | -0.59 to -0.41 |
| Impervious x Grass | | 0.42 | 0.11-0.75 |
| Lag 1 Temperature x Lag 2 Humidity | | -0.13 | -0.23 to -0.04 |
| **Parameter** | |  |  |
| Dispersion | | 2.27 | 2.00-2.63 |
| Spatial range | | 1.0 km | 0.6-1.7 km |
| **Indicator of Fit** |  | |  |
| DIC | 6306.60 | |  |

## Plots for Spatial Random Effect

We plotted the mean value of the spatial effect across the priority boundary area, along with its SD **(Appendix Figure 6)**. We noted that for *Aedes aegypti*, several traps in the southwestern part of the city had a higher spatial effect, while for *Aedes albopictus* traps in the south and eastern part of the downtown area had a higher spatial effect. The low spatial range resulted in a spatial effect that did not provide information farther than approximately 1 km from the study area, which is reflected in the large standard deviations away from observation locations in panels B and D.


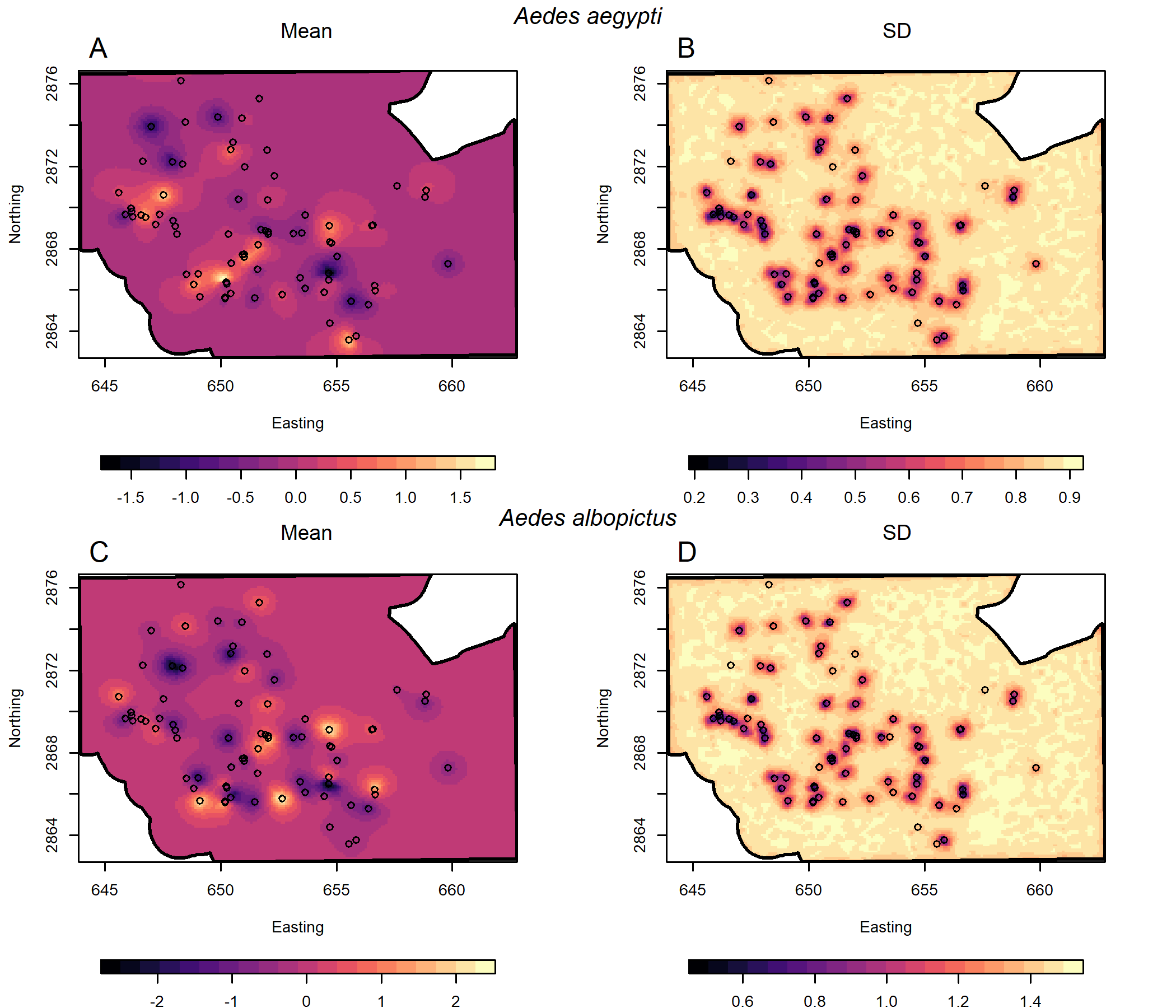


**Appendix Figure 6.** Mean and standard deviation of the spatial random effect for *Ae. aegypti* (A and B) and *Ae. albopictus* (C and D). Open circles represent mosquito trap locations. Brighter colors indicate a positive spatial effect, and a darker color indicates the opposite. The low range of the spatial effect (0.9 km and 1.4 km respectively) led to a relative lack of information about the spatial effect far from the trap locations. The axis labels refer to Northing and Easting kilometers in the NAD83/Zone 14N projected coordinate system.

## Weather Variable Plots

We plotted the values of weather variables from the DAYMET dataset (daily mean temperature, daily total precipitation, daily mean humidity, daily mean shortwave irradiance) averaged by week across the Brownsville priority area to aid in interpreting the predicted patterns of mosquito abundance. We note that the peak for shortwave irradiance coincides with the predicted peak of *Aedes aegypti* abundance, while temperature coincides with the observed peak. This may indicate the presence of an unmeasured variable such as vegetative biomass that we were unable to include in the model, causing a misspecification of the effects of temperature and irradiance. Future work will investigate this discrepancy.


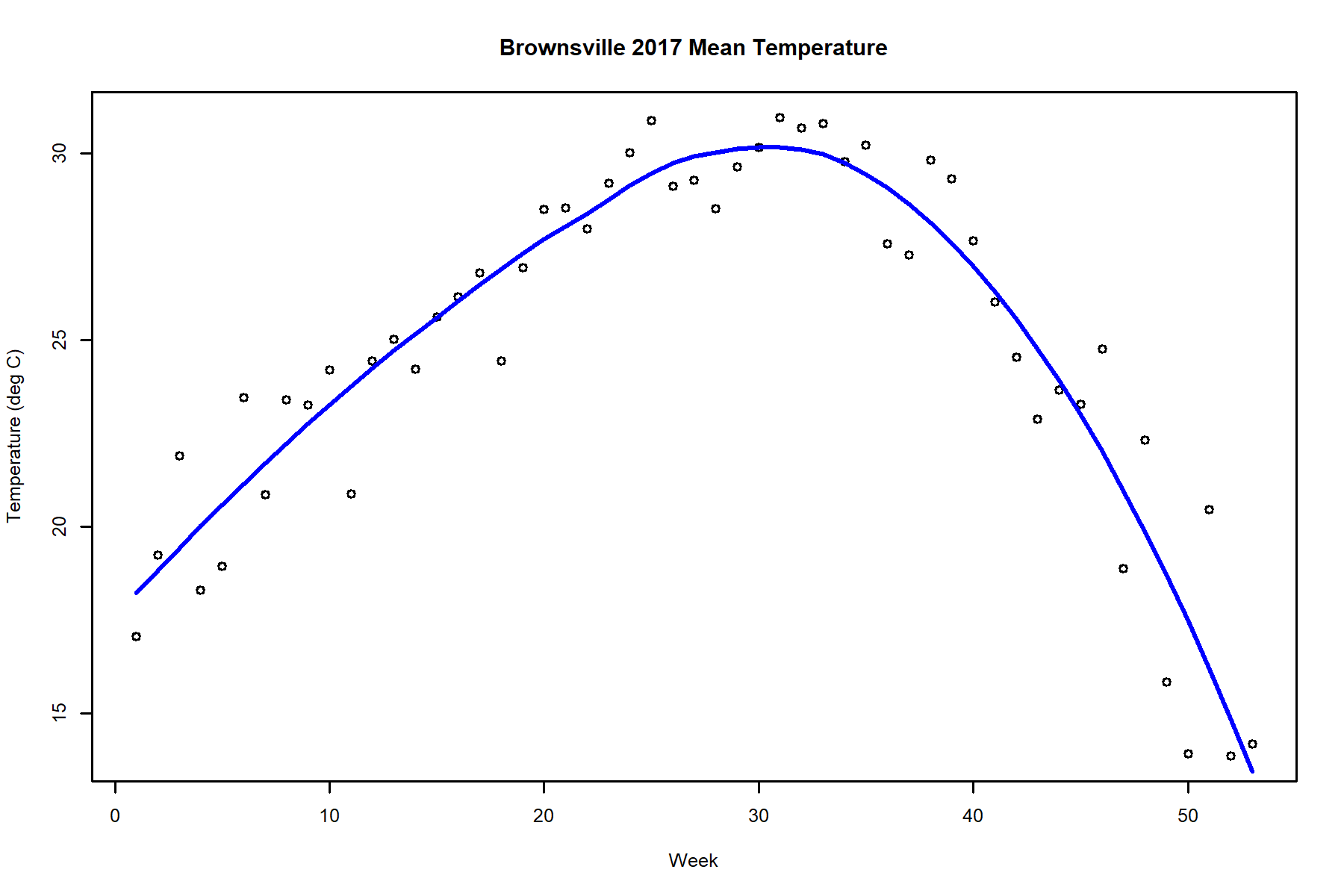


**Appendix Figure 7.** Mean daily temperature averaged by week across the Brownsville study area, from the DAYMET dataset. The blue line is a loess curve to aid in visualization of the trend over time.


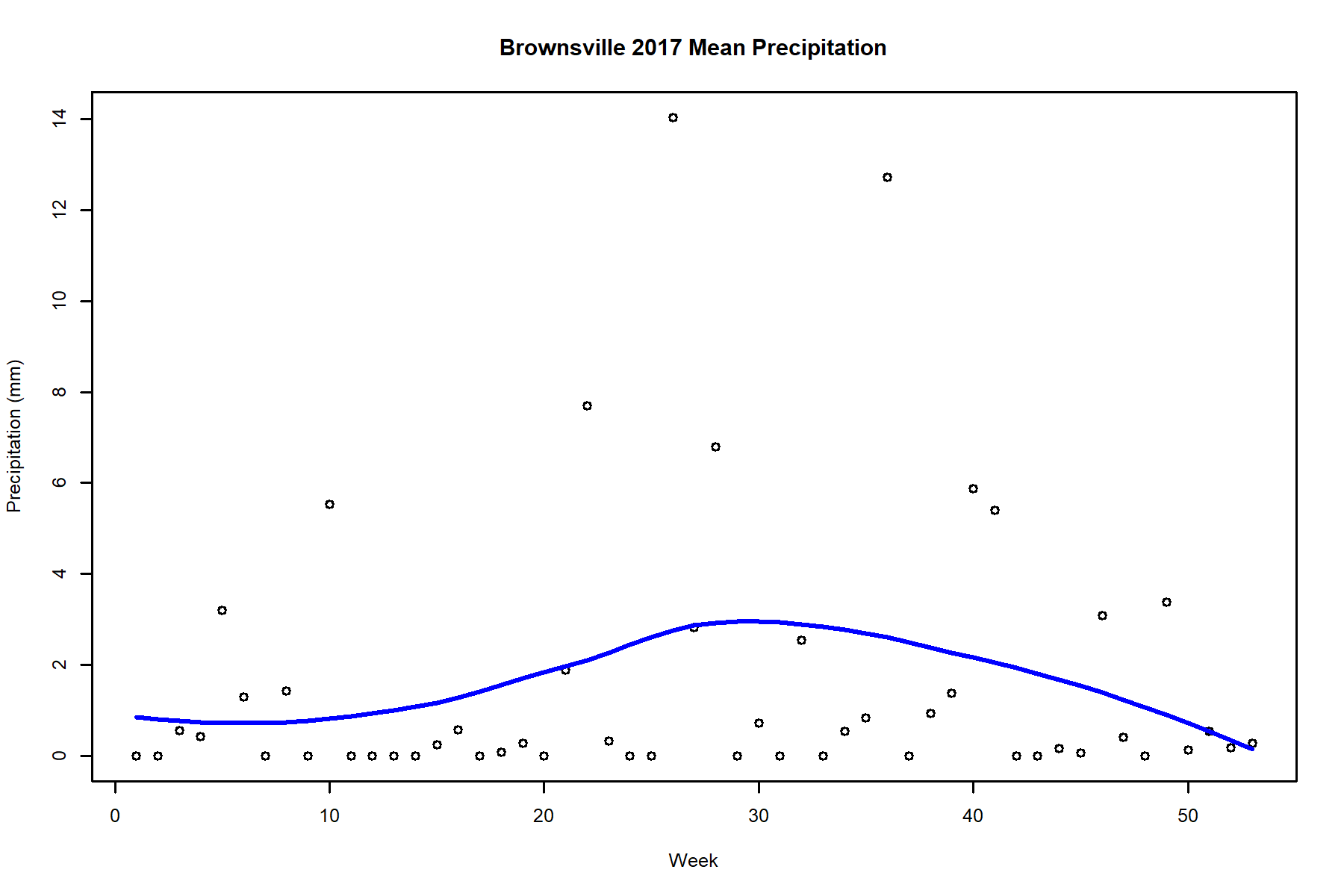


**Appendix Figure 8.** Total daily precipitation averaged by week across the Brownsville study area, from the DAYMET dataset. The blue line is a loess curve to aid in visualization of the trend over time.


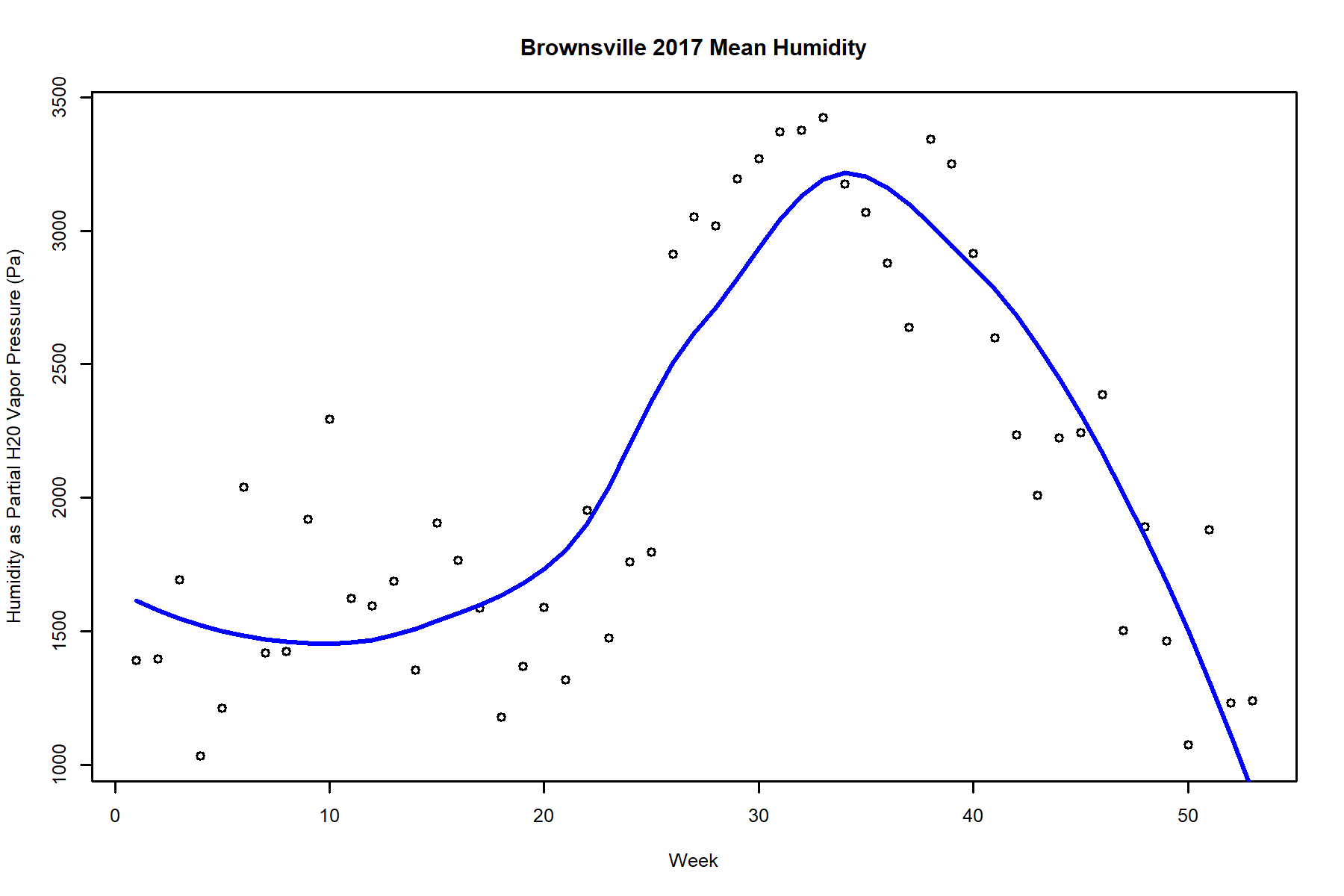


**Appendix Figure 9.** Mean daily humidity averaged by week across the Brownsville study area, from the DAYMET dataset. The blue line is a loess curve to aid in visualization of the trend over time.


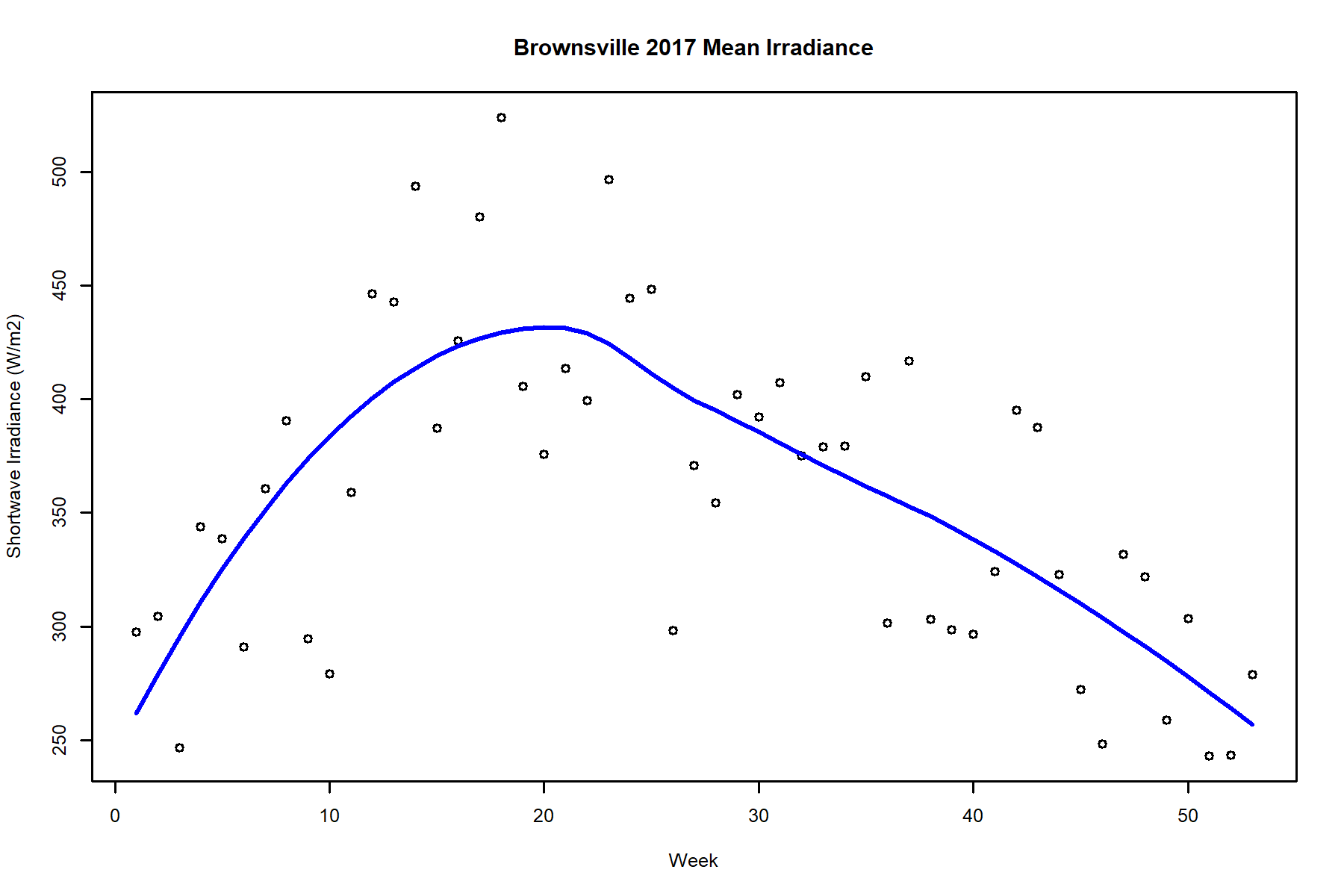


**Appendix Figure 10.** Mean daily shortwave irradiance averaged by week across the Brownsville study area, from the DAYMET dataset. The blue line is a loess curve to aid in visualization of the trend over time.
